# Unveiling the link between lactate metabolism and rheumatoid arthritis through integration of bioinformatics and machine learning

**DOI:** 10.1038/s41598-024-59907-6

**Published:** 2024-04-22

**Authors:** Fan Yang, Junyi Shen, Zhiming Zhao, Wei Shang, Hui Cai

**Affiliations:** Department of Chinese Medicine, Jinling Hospital, Affiliated Hospital of Medical School, Nanjing University, Nanjing, 210002 China

**Keywords:** Rheumatoid arthritis, Lactate metabolism, Immune infiltration, Bioinformatics analysis, Machine learning, Computational biology and bioinformatics, Genetics, Immunology

## Abstract

Rheumatoid arthritis (RA) is a persistent autoimmune condition characterized by synovitis and joint damage. Recent findings suggest a potential link to abnormal lactate metabolism. This study aims to identify lactate metabolism-related genes (LMRGs) in RA and investigate their correlation with the molecular mechanisms of RA immunity. Data on the gene expression profiles of RA synovial tissue samples were acquired from the gene expression omnibus (GEO) database. The RA database was acquired by obtaining the common LMRDEGs, and selecting the gene collection through an SVM model. Conducting the functional enrichment analysis, followed by immuno-infiltration analysis and protein–protein interaction networks. The results revealed that as possible markers associated with lactate metabolism in RA, KCNN4 and SLC25A4 may be involved in regulating macrophage function in the immune response to RA, whereas GATA2 is involved in the immune mechanism of DC cells. In conclusion, this study utilized bioinformatics analysis and machine learning to identify biomarkers associated with lactate metabolism in RA and examined their relationship with immune cell infiltration. These findings offer novel perspectives on potential diagnostic and therapeutic targets for RA.

## Introduction

Rheumatoid arthritis (RA), a systemic autoimmune disorder, is clinically distinguished by cartilage and bone destruction, frequently leading to disability and a reduced lifespan^1^. The global prevalence of RA is estimated to be 0.3–1%, with a male-to-female ratio of 1:6. The RA occurrence rate is approximately 0.3–0.5% in the Asia–Pacific region. The region’s significant population poses a considerable challenge regarding the economic burden of RA and the utilization of healthcare resources^2^. Different immune cells, such as synovial fibroblasts, monocytes, macrophages, and dendritic cells, may infiltrate and undergo stimulation for proliferation and differentiation due to the continuous CD4+ T cell growth during the initial phase of RA. This process produces numerous pro-inflammatory, chemokine, and angiogenesis factors^3^. A recent examination of the literature in this field discovered that lactate has been recognized as a possible indicator for RA^4^. Lactate may function as an active substance in RA patients with significant infiltration of lymphoid cells in their synovium, causing a shift in CD4+ T cells towards a pro-inflammatory state and exacerbating the disease^5^. Lactate is predominantly generated in the cytoplasm due to hypoxia or the increased glycolysis rate in rapidly dividing cells. The accumulated lactate is carried to the surrounding area, where it has the potential to enter various cells, including CD4+ T cells, macrophages, dendritic cells, and osteoclasts. Lactate has two possible effects. On the one hand, lactate is preferred by active immune cells as a means of supporting their activity. Conversely, the build-up of lactate inside the tissue microenvironment functions as a signaling molecule that limits the ability of immune cells to function. Therefore, the target cells may undergo differentiation and activation, impacting their performance and ultimately contributing to RA development^6,7^. Nonetheless, the precise molecular process of lactate metabolism and the infiltration of immune cells in RA remains uncertain. Hence, the quest for biomarkers holds immense significance for identifying and treating RA using immunotherapy.

A growing body of research has concentrated on the crucial significance of immune infiltration in RA progression. Most of the inflammatory infiltration in RA is composed of the synovial subliming's myeloid pathotype, including monocytes and/or macrophages. Positive correlations exist between the extent of macrophage infiltration in joint tissues and cytokine levels derived from monocytes in the bloodstream^8^. Additionally, identifying genes associated with RA diagnosis relies heavily on bioinformatics analysis and machine learning techniques. A prior bioinformatics investigation revealed that CLP1 could substantially impact RA's progression by modifying immune cell infiltration^9^. The potential usefulness of LSP1, GNLY, and MEOX2 in diagnosing and treating RA, along with the potential influence of immune cell infiltration on the development and advancement of RA, should not be underestimated^10^. A recent investigation discovered that GZMA-Tfh cells, CCL5-M1 macrophages, and CXCR4- memory activated CD4+ T cells/Tfh cells could potentially affect the development and advancement of RA, with particular emphasis on GZMA-Tfh cells during the initial stages of RA pathogenesis^11^. However, lactate metabolism and the molecular processes underlying immune cell infiltration in RA are poorly understood. Further examination of immune cell infiltration and exploration of potential therapeutic targets linked to it are necessary.

The study utilized a microarray dataset of synovial tissue from individuals with RA and without health issues acquired from the GEO database. The dataset was used to screen genes related to lactate metabolism. Additionally, bioinformatics analysis and machine learning, using two algorithms, namely CIBERSORTx and ssGSEA, were employed to perform immune infiltration analysis. The objective was to identify disparities in immune cell infiltration and potential biomarkers and explore the connection between immune cells and lactate metabolism-related genes and the role of lactate metabolism in immune cell infiltration during RA progression.

## Methods

### Data download

RA-related datasets GSE1919^12^, GSE29746^13^ and GSE55235^14^ from the GEO database^15^ were obtained using the R package GEOquery^16^. The data platform for GSE1919 was GPL91 [HG_U95A] Affymetrix Human Genome U95A Array, and it included microarray gene expression profiling data of synovial tissue samples from five patients with RA (RA group) and five fully matched normal subjects (Control group). The data platform for GSE29746 was GPL4133 Agilent-014850 Whole Human Genome Microarray 4x44K G4112F (Feature Number version), originating from *Homo sapiens*. Synovial tissue samples were chosen from nine patients diagnosed with RA and 11 partially matched samples. The gene expression profile data of synovial tissue samples from individuals without abnormalities served as the Control group. The data platform for GSE55235 was GPL14951 Illumina HumanHT-12 WG-DASL V4.0 R2 expression bead chip GPL96 [HG-U133A] Affymetrix Human Genome U133A Array. It consisted of microarray gene expression profile data from synovial tissue samples of 10 RA patients (RA group) and synovial tissue samples of 10 completely matched normal subjects (Control group) from *H. Sapiens*. This study included all the samples that were selected. The annotation of the dataset probe name utilizes the associated GPL platform file. Table 1 displays the dataset details.

**Table 1:** Information of datasets.

Items	GSE1919	GSE29746	GSE55235
Platform	GPL91	GPL4133	GPL96
Sorting type	Expression profiling by array	Expression profiling by array	Expression profiling by array
Species	Homo sapiens	Homo sapiens	Homo sapiens
Disease	RA	RA	RA
Tissue	Synovial tissue	Synovial tissue	Synovial tissue
Samples in disease group	5	9	10
Samples in control group	5	11	10
Reference	20858714	22021863	24690414

RA, rheumatoid arthritis.

The GeneCards database^17^ offers thorough details on human genes. The phrase ‘lactate metabolism’ was employed as our search term to retrieve lactate metabolism-related genes (LMRGs) from the GeneCards database. After filtering LMRGs that were unclassified as ‘Protein Coding’ and had a ‘Relevance score’ greater than 2, two LMRGs were successfully identified. Furthermore, associated pathways containing the keyword ‘autophagy’ were obtained from the Molecular Signatures Database (MSigDB), and 289 LMRGs from eight gene sets considered references were compiled. In this study, the LMRGs obtained from the two sources were combined, resulting in 289 LMRGs available for analysis. Table S1 presents precise gene designations.

### Differential expression analysis

To identify the likely biological functions, characteristics, and pathways of the different genes between the RA disease and control groups. Initially, the RA datasets GSE1919, GSE29746, and GSE55235 underwent batch effect removal to obtain the merged RA dataset. Then, the data sets were compared before and after the batch effect removal using distribution boxplots and principal component analysis (PCA) graphs. The RA dataset was split into the RA and Control groups for differential analysis. Differential expression genes (DEGs) were identified using *P* < 0.05 and |logFC|> 0 thresholds. Genes with logFC > 0 and *P* < 0.05 were considered up-regulated differentially expressed, while genes with logFC < 0 and P < 0.05 were considered down-regulated differentially expressed. The R package ggplot2 was used to create a volcano map, displaying the outcomes of the differential analysis. For subsequent analysis, lactate metabolism-related differential expression genes (LMRDEGs) were obtained by intersecting DEGs with LMRGs. Next, a comparison graph was created to analyze the grouping differences between the RA and Control groups in the RA dataset for LMRDEGs. Then, the key genes were identified for further analysis based on their statistically significant differences. The R package RCircos^17^ was employed to create a chromosome map and visualize the essential gene arrangement on human chromosomes. Predicting possible functional similarity by chromosome distribution. Additionally, the R package pheatmap was utilized to represent gene expression as a heatmap visually.

### Support vector machines (SVM) screening model

SVM^18^ represents a model for classifying data into two categories. The fundamental design is a linear classifier with the widest range defined within the feature space. A model was constructed utilizing the SVM algorithm and LMRDEGs as the basis. The primary genes for the subsequent analysis were selected based on their precision, with preference given to those with the highest (lowest error rate) number.

### Gene Ontology (GO) and Kyoto Encyclopedia of Genes and Genomes (KEGG)

GO analysis is commonly utilized in large-scale studies to investigate functional enrichment, encompassing biological process (BP), molecular function (MF), and cellular components (CC)^19^. The extensively utilized KEGG^20^ database encompasses information regarding genomes, biological pathways, diseases, drugs, and various other subjects. The clusterProfiler R package was utilized^21^ to conduct GO and KEGG annotation analysis on the pivotal genes. The entry screening criteria included a P-value less than 0.05 and a false discovery rate (FDR) value (q-value) less than 0.25 to be considered significantly enriched. The correction method for the P-value was BH (Benjamini-Hochberg).

### Gene set enrichment analysis (GSEA)

The gene table was arranged based on their connection with the phenotype to determine the contribution of genes to the phenotype. GSEA^22^ was employed to evaluate the distribution pattern of the genes in a predefined gene set. The gene set ‘c2.cp.all.v2022.1.Hs.symbols’ was acquired from the MSigDB database^23^. Subsequently, the R package clusterProfiler was employed to examine the RA and Control groups within the RA dataset. The GSEA was performed on all genes using the following parameters: the seed was set to 2022, 100,000 calculations were performed, and each gene set contained a minimum of five genes and a maximum of 500 genes. The P-value correction method was BH, and the significant enrichment was determined based on the criteria of *P* < 0.05 and FDR value (q.value) < 0.25.

### Gene set variation analysis (GSVA)

GSVA^24^ is an unsupervised and non-parametric technique that mainly involves transforming the expression matrix of specific genes across samples into the expression matrix of specific sets of genes. To assess the enrichment results of gene sets in the nuclear transcriptome microarray data. To evaluate if various pathways are enriched across distinct samples. The gene set ‘h.all.v7.4.symbols.gmt’ was acquired from the MSigDB database. GSVA was conducted on the RA dataset to assess the disparity in functional enrichment among the two sample groups based on gene expression levels. The set was screened based on the criterion that *P* < 0.05.

### Immune infiltration analysis

Based on the linear support vector regression theory, the CIBERSORTx algorithm was used to analyze immune infiltration and determine the composition and quantity of immune cells in mixed cell populations by deconvoluting the transcriptome expression matrix. After uploading the gene expression matrix data from the RA dataset to CIBERSORTx, it was combined with the LM22 characteristic gene matrix. After eliminating the data with an immune cell enrichment score above zero, the accurate outcomes of the matrix displaying the abundance of immune cell infiltration were obtained and showcased. The stacked histograms display and calculate the ratio of immune cell infiltration in various sample groups within the GDM dataset. The gene expression matrix of the data set was merged to compute the correlation between immune cells and important genes in various groups of the RA dataset. Subsequently, the R package ggplot2 was utilized to generate a correlation dot plot for visualization.

The proportionate prevalence of every immune cell infiltration was measured using the ssGSEA algorithm for single-sample gene-set enrichment analysis. Reflect the relative abundance of immune cell infiltration in each sample using the enrichment fraction acquired through ssGSEA. Label different types of invading immune cells, including CD8+ T lymphocytes, dendritic cells, macrophages, regulatory T lymphocytes, and other subcategories of human immune cells^25^. The overall infiltration level of 28 immune cells in each sample was represented using the enrichment score obtained from the analysis of the ssGSEA algorithm in the R package GSVA. The disparity and association of immune cell infiltration levels were examined between the two algorithms using RA and Control groups (or other grouping) and key genes. The outcomes were displayed in a group comparison chart, correlation heat map, and complex heat map.

### Protein–protein interaction (PPI)

PPI is a network of distinct proteins that interact with one another. The STRING database^26^ identifies proteins and predicts their interactions. For this research, a PPI network was generated using the STRING database (with a minimum interaction score of 0.150) based on the identified hub genes. Chemical complexes with specific biological functions may exist within the interconnected sections of the PPI network. Consequently, genes were identified in the PPI network interacting with other central genes and included in the subsequent analyses. Visual PPI network models were constructed using Cytoscape software^27^ (version 3.9.1). The GeneMANIA website^28^ was utilized to predict genes with similar functions to the target genes. The GeneMANIA website was utilized to construct networks of interactions and make predictions about hub genes.

### Prediction networks for RNA-miRNA, mRNA-TF, mRNA-drug, mRNA-RBP

ENCORI, a database^29^, offers a high-throughput search for miRNA targets using CLIP-Seq and degradome techniques. It presents diverse visualization interfaces to explore miRNA targets and encompasses extensive data on miRNA-lncRNA, miRNA-mRNA, miRNA-RNA, and RNA-lncRNA interactions. The ENCORI database was utilized to predict miRNAs that interact with CRRDEGs. Subsequently, the results were filtered to include only miRNAs with a database number above three. The mRNA-miRNA interaction network was visualized using the Cytoscape software. The ENCORI database was utilized to predict RBPs that interact with CRRDEGs. Subsequently, RBPs with shear fragments greater than five in upstream and downstream regions were selected from the results to construct the mRNA-RBP interaction network using Cytoscape software.

The CHIPBase database^30^ (version 3.0) (https://rna.sysu.wsu.cn/chipbase/) was used to discover numerous binding motif matrices and their corresponding binding sites from the ChIP-seq data of DNA-binding proteins. Additionally, it predicted millions of transcription factors (TF) and gene transcriptional regulation. After utilizing the CHIPBase database to predict TFs interacting with CRRDEGs and filtering for TFs with over 14 supporting samples, the mRNA-TF interaction network was constructed using Cytoscape software.

The DGIdb database^31^, also known as the drug-gene interaction database, consolidates documented drug-gene interactions from various sources, including DrugBank, PharmGKB, Chembl, Drug Target Commons, and TTD, along with literature reports. The platform offers two categories of information: documented drug-gene interactions derived from literature sources and anticipated drug-gene interactions projected through analysis of functional, structural, and other attributes shared between drugs or gene families. Drugs interacting with CRRDEGs were filtered for medications with more than two reference counts using the DGIdb database. Subsequently, Cytoscape was employed to visualize the mRNA-drug interaction network.

### Statistical analysis

R software (version 4.2.2) was used to perform all data processing and analysis in this study. The Wilcoxon rank sum test was used to compare two groups of continuous variables, and the independent student t-test was used to estimate the statistical significance of normally distributed variables. The Kruskal–Wallis test was utilized to compare involving three or more groups. Fisher’s exact or chi-square test was employed to assess and compare the statistical significance of two sets of categorical variables. The outcomes were computed using Spearman rank correlation analysis if not explicitly stated. The correlation coefficient was determined between diverse molecules or scores; All *P* statistics were considered two-sided. A *P*-value below 0.05 was considered the threshold for statistical significance. The figures in graphical abstract were produced by Figdraw and Adobe illustrator (version 26.0).

## Results

### Technology roadmap

**Figure 1:**
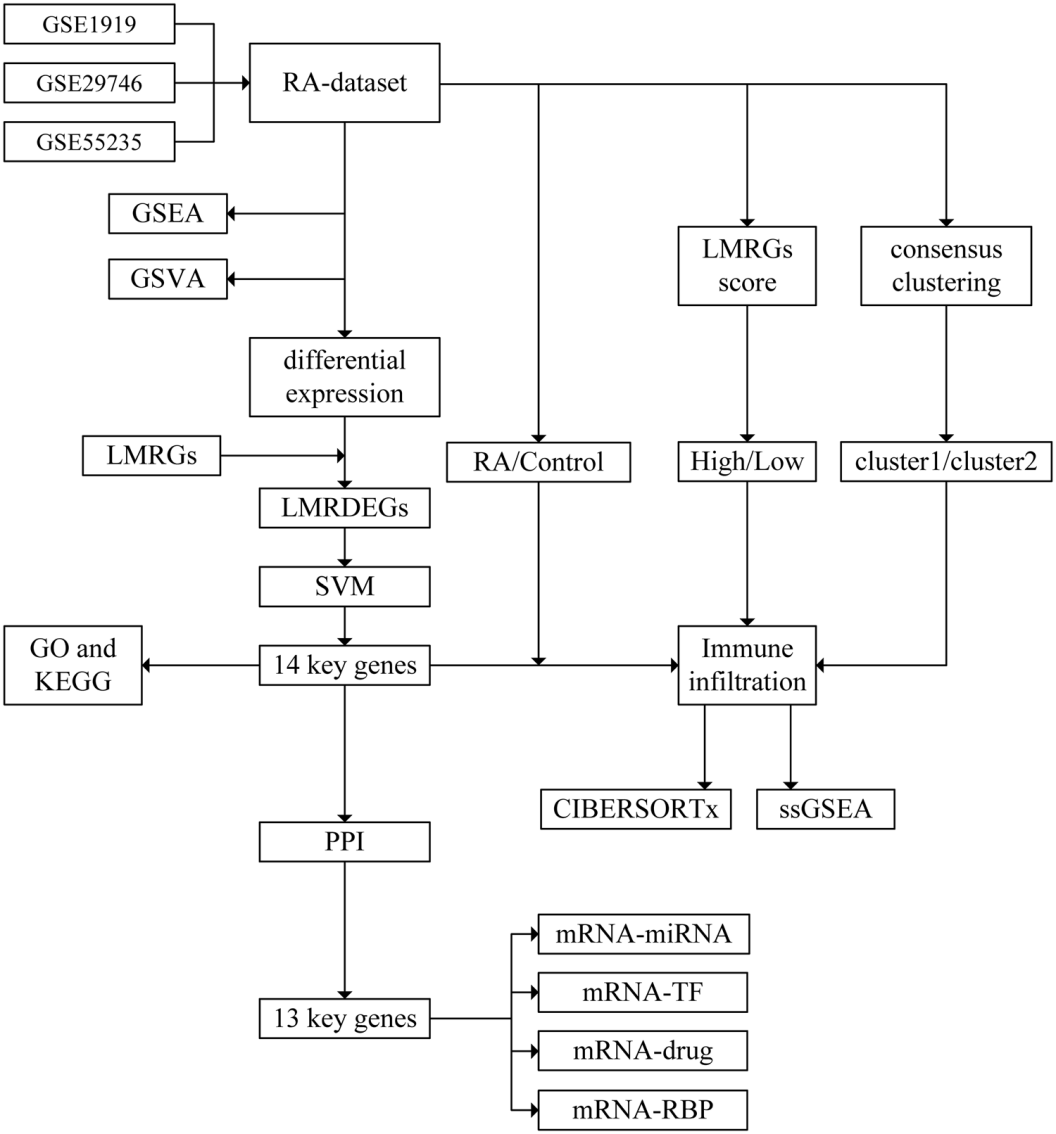
Flow chat. RA, rheumatoid arthritis. LMRGs, lactate metabolism related genes. LMRDEG, lactate metabolism related differential expression genes. GO, gene ontology. KEGG, Kyoto encyclopedia of genes and genomes. GSEA, gene set enrichment analysis. GSVA, gene set variation analysis. ssGSEA, single-sample gene set enrichment analysis. PPI, protein–protein interaction. TF, transcription factor. RBP, RNA binding protein.

Figure 1 displays the flowchart. Initially, the GSE1919, GSE29746, and GSE55235 datasets related to RA were subjected to batch effect removal. Subsequently, the combined RA dataset was obtained and analyzed to compare the RA group with the Control group. DEGs and LMRGs meeting the |logFC|> 0 and *P* < 0.05 criteria were screened and intersected to derive LMRDEGs. Graphs presented the comparison, we analyzed the chromosomal location and functional similarity of important genes, conducting correlation analysis on these gene expressions in the RA dataset. The crucial genes were analyzed using GO and KEGG methods. Subsequently, GSEA, GSVA, and immune infiltration analysis were performed on all samples in the RA dataset using two algorithms, CIBERSORTx and ssGSEA. Next, we utilize the crucial genes in the RA dataset to create the LMRGs score for the samples. Subsequently, we categorize the RA group samples into the High and Low groups based on the phenotype score median. Finally, we analyzed immune infiltration using CIBERSORTx and ssGSEA algorithms on this categorized data. Next, we utilized crucial genes to establish disease subcategories within the RA group of the RA dataset. Then, the outcomes were divided into two clusters: cluster1 and cluster2. Subsequently, we conducted immune infiltration analysis in this group using CIBERSORTx and ssGSEA, two algorithms. We construct the PPI network by selecting the essential genes from the STRING database with a confidence threshold 0.150. We input the protein genes that interact with other genes into the GeneMANIA database. Finally, we gathered information from the ENCORI database to create the mRNA-miRNA and mRNA-RBP interaction networks for important genes. Additionally, we utilized data from the ChIPBase3.0 database to construct the mRNA-TF interaction network, and obtained data from the DGidb database to establish the mRNA-drug interaction network for key genes.

### Variations in the manifestation of LMRGs within the RA dataset

Initially, the RA datasets GSE1919, GSE29746, and GSE55235 underwent batch effect removal processing, yielding the merged data set RA dataset (supplementary Fig. S1).

**Figure 2:**
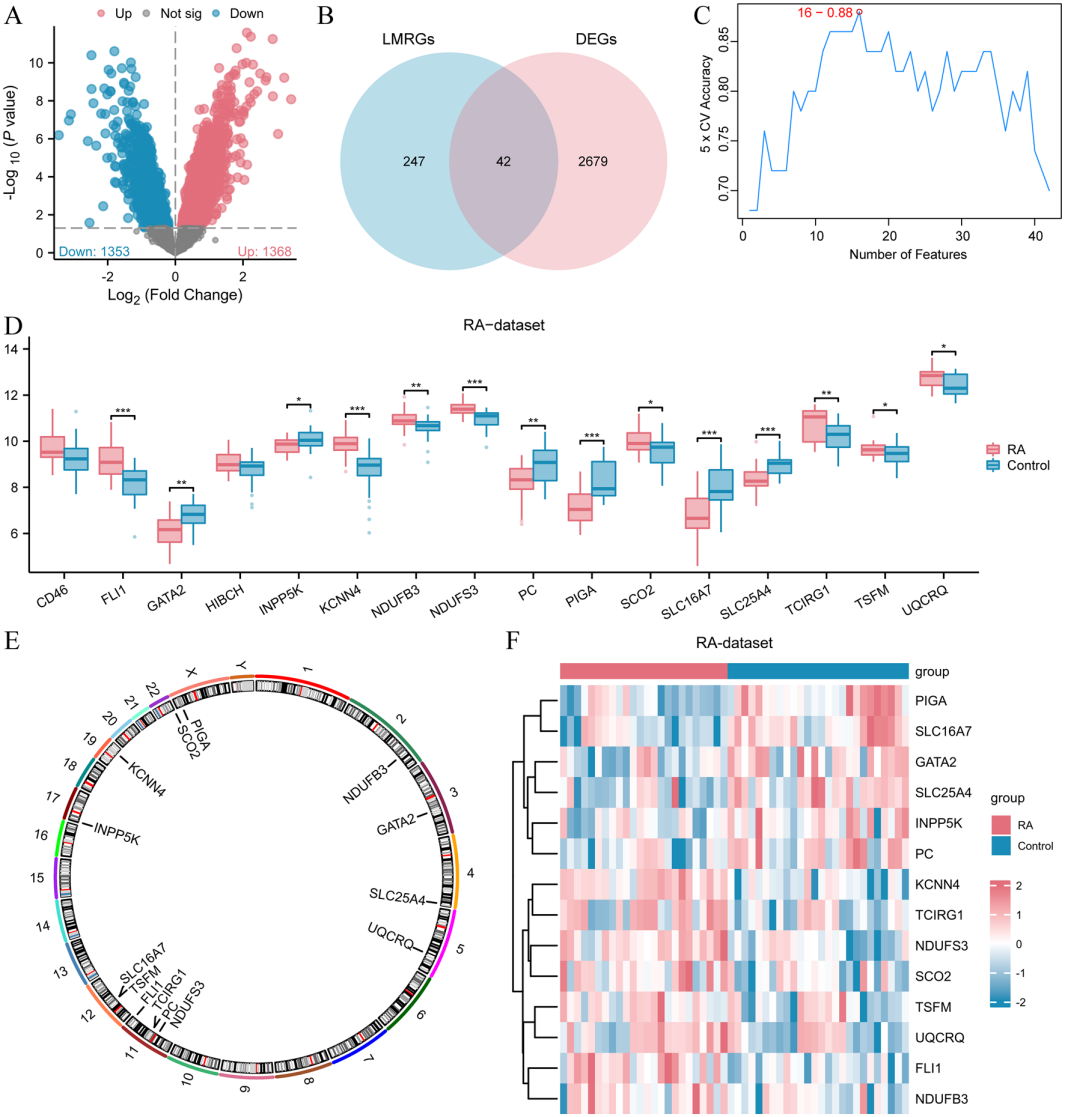
Expression difference of LMRGs in RA dataset. (**A**) Volcano plots showing changes in gene expression in the RA-dataset. The horizontal axis is the log2 fold change and the vertical axis is the negative log10 *P*-value. Up-regulated genes (blue) and down-regulated genes (red) are delimited by a horizontal dashed line (*P*-value threshold) and two vertical dashed lines (fold change threshold). The figure shows a total of 1368 up-regulated genes and 1353 down-regulated genes. (**B**) Venn diagram illustrating the overlap between differentially expressed genes and LMRGs. (**C**) SVM model screening LMRDEGs display. (**D**) A comparison chart presents LMRDEGs in the RA dataset. Chromosomal map of (**E**) key genes. (**F**) The RA dataset contains a heat map displaying the important gene expressions. The * symbol in the group comparison chart (CD) represents a statistical significance of *P* < 0.05. The ** symbol represents a high statistical significance of *P* < 0.01. The *** symbol represents a very high statistical significance of *P* < 0.001, indicating significant meaning. LMRG, lactate metabolism-related genes; DEGs, differential expression genes. LMRDEG, lactate metabolism-related differential expression genes; and RA: rheumatoid arthritis.

A total of 2,721 genes satisfied the | logFC |> 0 criteria and *P* < 0.05. Among these genes, 1368 had high expression in the RA group, while the remaining 1353 genes had low expression in the RA group. We generated a volcano map (Fig. 2A) to visualize the differential analysis results of the RA dataset. We successfully identified 42 LMRDEGs by comparing the acquired genes expressed differently with LMRGs. Additionally, a Venn diagram (Fig. 2B) was created to represent the intersection visually. We screened key genes from the RA dataset using SVM. The model results (Fig. 2C) revealed 16 genes (CD46, FLI1, GATA2, HIBCH, INPP5K, KCNN4, NDUFB3, NDUFS3, PC, PIGA, SCO2. (SLC16A7, SLC25A4, TCIRG1, TSFM, UQCRQ). Next, we examined the variations in the expression levels for 16 LMRDEGs between the RA and Control groups within the RA dataset. Figure 2D presents the findings in a comparative chart. The findings indicated that 14 genes (FLI1, GATA2, INPP5K, KCNN4, NDUFB3, NDUFS3, PC, PIGA, SCO2, SLC16A7, SLC25A4, TCIRG1, TSFM, and UQCRQ) exhibit statistically significant variances between the two groups (*P* < 0.05). These 14 genes will be considered crucial genes in the subsequent analysis. Table S2 depicts detailed information about each gene. We annotated their positions and created a chromosome location map to examine the locations of these 14 crucial genes on human chromosomes (Fig. 2E). This map reveals that genes FLI1, NDUFS3, PC, and TCIRG1 are located on chromosome 11, while SLC16A7 and TSFM reside on chromosome 12. The remaining key genes are dispersed across various chromosomes. A heat map (Fig. 2F) was also generated to display the 14 crucial gene expressions in the RA dataset.

### Correlation analysis of key genes

The Spearman technique was used to analyze the 14 key gene expression levels in the RA group samples of the RA dataset. The findings indicated that the gene GATA2 in the RA dataset and the genes (SLC25A4, TCIRG1), PIGA, SLC16A7, TCIRG1, UQCRQ, KCNN4, and UQCRQ) exhibited a moderate positive linear correlation (*r* > 0.3, *P* < 0.05) (Supplementary Fig. S2A, B). Functional similarity analysis was employed to assess the functional similarity of key genes. The results were presented as a box plot based on the score (Supplementary Fig. S2C). The figure indicates that GATA2 has the highest functional similarity score. Additionally, we chose the top four gene pairs that exhibited the most robust positive linear correlation among the 14 essential genes. These pairs were used to visualize a correlation scatter plot (Supplementary Fig. S2D–G).

### GO and KEGG

**Figure 3:**
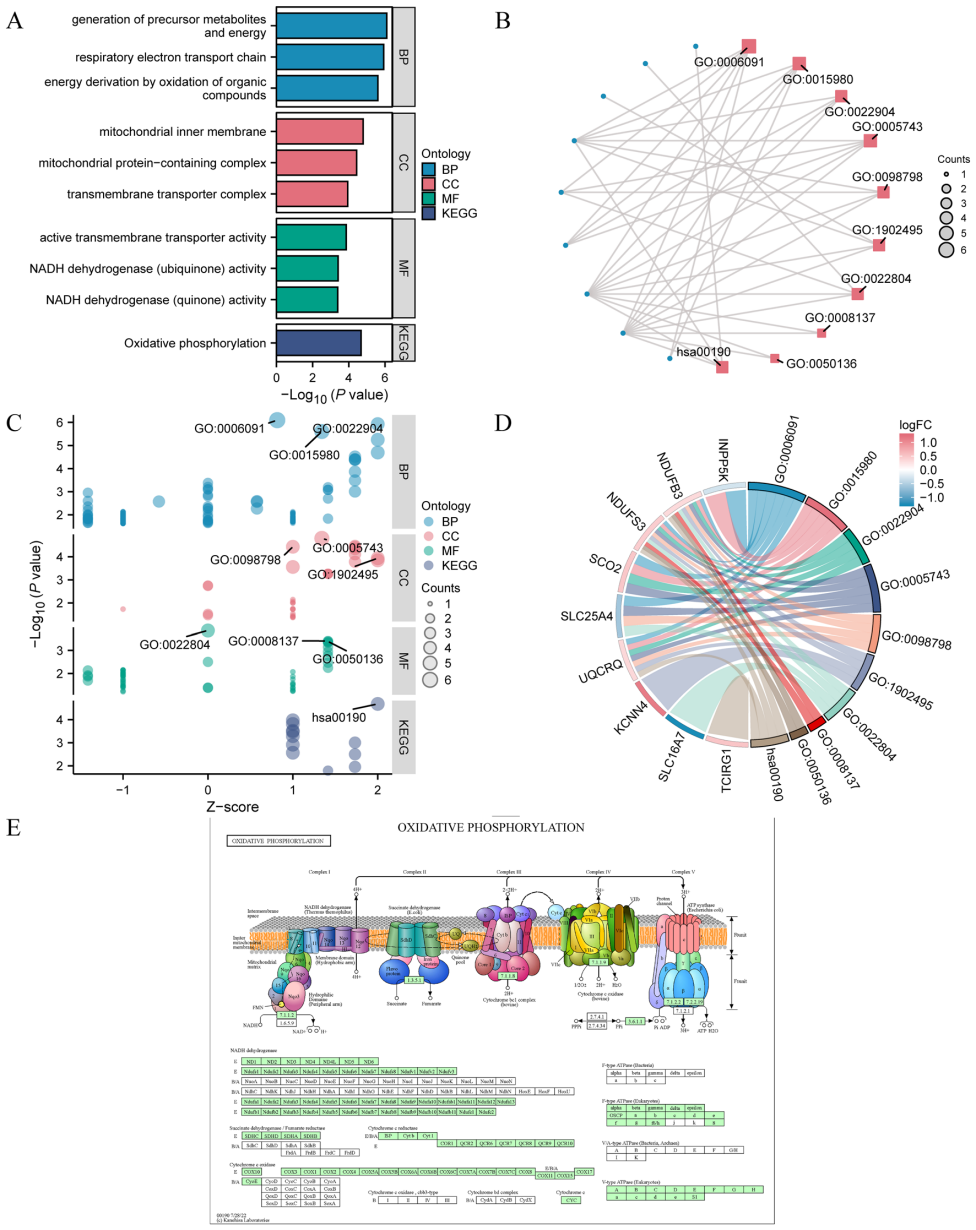
GO function enrichment and KEGG pathway enrichment analysis. AB. The histogram (**A**) and network diagram (**B**) illustrate the GO and KEGG enrichment analysis results for the key genes. The enrichment analysis results for GO and KEGG are based on the combined logFC. CD. Bubble plot (**C**) and chord plot (**D**) display the identified crucial genes. (**E**) Necroptosis KEGG pathway diagram (hsa04217). The pathway diagrams of E are obtained by downloading them from the KEGG Pathway database. The screening criteria included a significance level of P < 0.05 and an FDR value (q-value) below 0.25 to qualify for GO and KEGG enrichment. GO, Gene Ontology; BP, biological process. CC, cellular component; MF, molecular function; and KEGG, Kyoto encyclopedia of genes and genomes.

Initially, we conducted GO gene function enrichment analysis on 14 genes to examine the biological processes, molecular functions, cellular components, and biological pathways associated with 14 specific genes about RA (Supplementary Table S3). The enrichment entries were screened based on having a *P*-value less than 0.05 and an FDR value (q-value) less than 0.25. The findings indicate that the 14 main genes are primarily concentrated in the biological process of producing precursor metabolites and energy (GO 0006091), deriving energy through the oxidation of organic compounds (GO 0015980), the respiratory electron transport chain (GO 0022904), and other biological processes in RA. Regarding cellular components, they are found in the mitochondrial inner membrane (GO 0005743), mitochondrial protein-containing complex (GO 0098798), transmembrane transporter complex (GO 1902495), and other biological processes. Furthermore, regarding molecular functions, they exhibit active transmembrane transporter activity (GO 0022804), NADH dehydrogenase (ubiquinone) activity (GO 0008137), NADH dehydrogenase (quinone) activity (GO 0050136), and other molecular functions. Afterward, KEGG enrichment analysis was conducted on 14 important genes (Supplementary Table S3). The findings indicated significant enrichment of 14 crucial genes in KEGG pathways, including Oxidative phosphorylation (hsa00190). The histogram (Fig. 3A) and divergence network diagram (Fig. 3B) displayed GO and KEGG enrichment analysis outcomes. Next, we combined logFC GO and KEGG enrichment analysis on 14 pivotal genes. The bubble diagram (Fig. 3C) and the chord diagram (Fig. 3D) displayed the GO and KEGG enrichment analysis results for the joint logFC. Additionally, the pathway diagram depicted the KEGG pathway Oxidative phosphorylation (hsa00190) (Fig. 3E).

### GSEA

**Figure 4:**
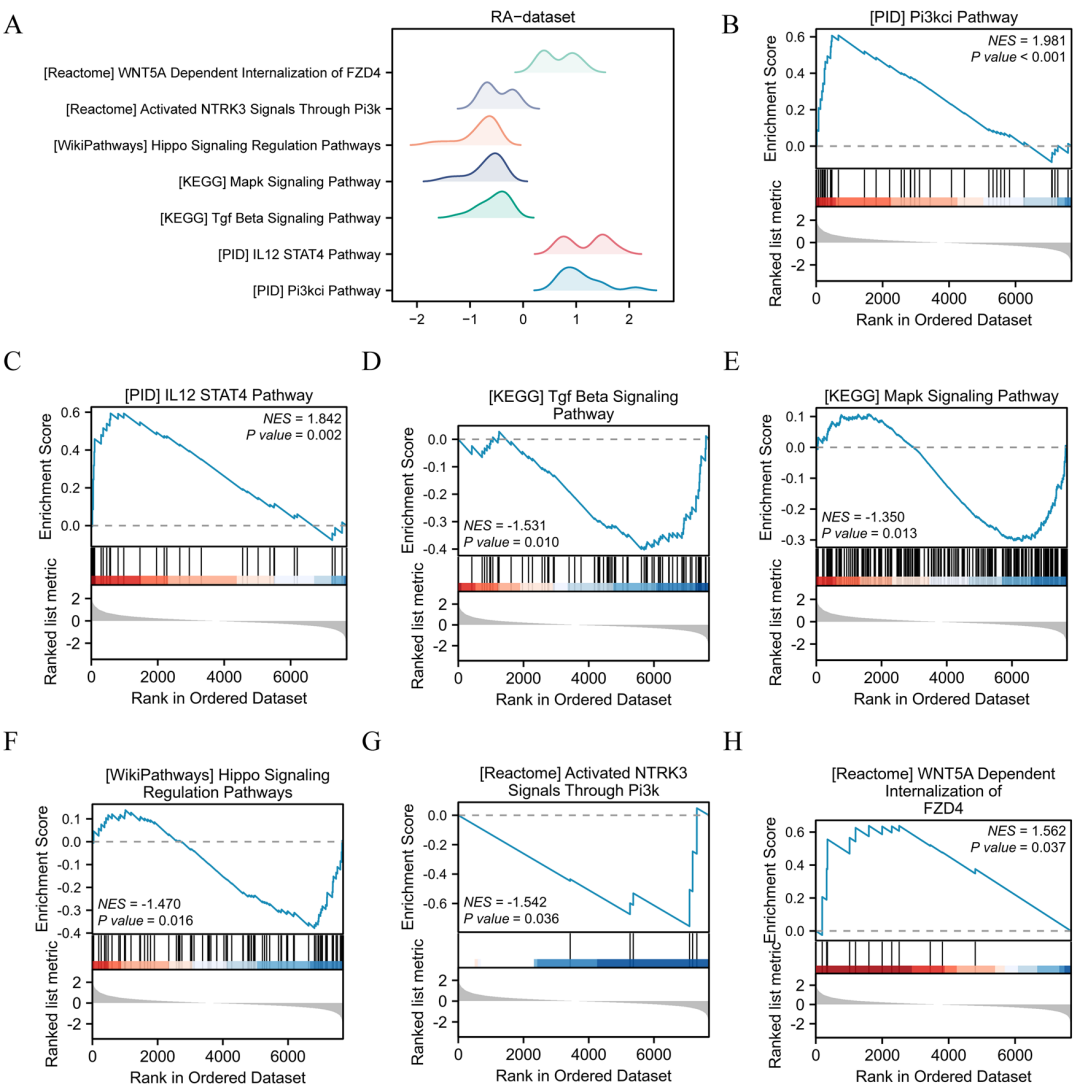
Gene sets enrichment analysis (GSEA). (**A**) Enrichment distribution curves for a range of biological pathways are shown at the top. These curves depict the ranked distribution of genes in the examined biological pathways in the RA-dataset dataset. We can see the trend of enrichment in the dataset for different pathways such as WNT5A-dependent FZD4 internalisation, activated NTRK3 via PI3k signalling, Hippo signalling regulatory pathway, Mapk signalling pathway, Tgf Beta signalling pathway, IL12 STAT4 pathway and PI3ki pathway. (**B**–**H**) The RA dataset contains genes that are notably enriched in the PI3KCI pathway (**B**), IL12 STAT4 pathway (**C**), TGF-β signaling pathway (**D**), MAPK signaling pathway (**E**), HIPPO signaling regulation pathways (**F**), activated NTRK3 signals via PI3K (**G**), WNT5A dependent internalization of FZD4 (**H**), and various other pathways. The important criteria for GSEA enrichment screening were a *P*-value less than 0.05 and an FDR value (q-value) less than 0.25. RA, rheumatoid arthritis; GSEA, Gene sets enrichment analysis.

We conducted GSEA to examine the influence of gene expression levels on the disparities between the RA and Control groups in RA. A significance level of P < 0.05 and a FDR value (q-value) < 0.25 were employed as the criteria for significant enrichment to establish the relationship between functions (Supplementary Table S4). In the mountain map (Fig. 4A) and the pathway map (Figs. 4B–H), we present the significantly enriched pathways, including the PI3KCI pathway (Fig. 4B), IL12 STAT4 pathway (Fig. 4C), TGF-β SIGNALING pathway (Fig. 4D), MAPK signaling pathway (Fig. 4E), HIPPO signaling regulation pathways (Fig. 4F), activated NTRK3 signals via PI3K (Fig. 4G), and WNT5A dependent internalization of FZD4 (Fig. 4H), containing star hotspot molecules relevant.

### GSVA

**Figure 5:**
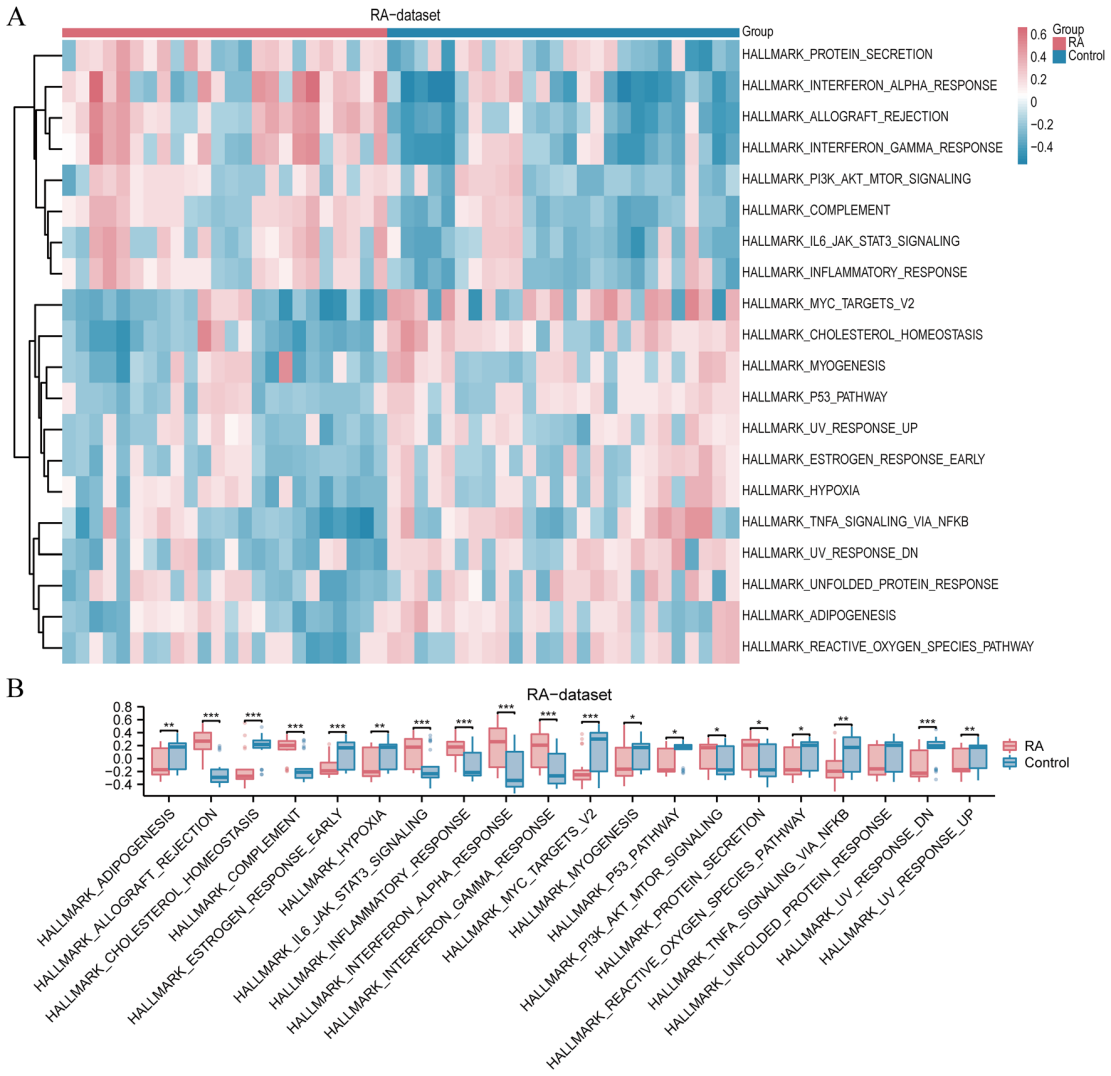
Analysis of variations in gene sets. (**A**) The Heatmap showing the expression of different sets of genes in different samples. Each column represents one sample, grouped into RA (rheumatoid arthritis) and control groups. Each row represents a gene set such as “HALLMARK_INTERFERON_GAMMA_RESPONSE” (interferon-gamma response) or “HALLMARK_HYPOXIA” (hypoxia). Colors represent Z-scores: pink represents higher gene set activity (positive Z-scores) and blue represents lower gene set activity (negative Z-scores). The clustering tree (dendrogram) on the left side of the heatmap represents the similarity between gene sets, where similar gene sets are grouped together. (**B**) The box plots show the differences in the activity of some key sets of genes in the RA and control groups. Red box plots represent the RA group and blue represents the control group. In each pair of box plots, the centre line of the box indicates the median, the range of the box indicates the first and third quartiles, and the tentacles indicate the range of outliers. The primary screening criterion for GSVA enrichment analysis was a significance level of less than 0.05. In the group (**B**) comparison chart, the symbol ns represents *P* ≥ 0.05, indicating no statistical significance. The symbol * represents *P* < 0.05, indicating statistical significance. The symbol ** represents *P* < 0.01, indicating high statistical significance. The symbol *** represents a *P*-value < 0.001, indicating very high statistical significance. RA, rheumatoid arthritis; GSVA, Gene set variation analysis.

Subsequently, we conducted GSVA on the gene expression data of all genes in the RA dataset to investigate the variation in the characteristic gene set between the RA and Control groups (Supplementary Table S5). The GSVA findings indicated variations in 20 hallmark gene sets between the RA and Control groups (*P*-value < 0.05, as depicted in Fig. 5A). We created a comparative chart (Fig. 5B) for 20 characteristic gene sets to illustrate the variations in expression levels. The analysis revealed statistically significant differences (*P*-value < 0.05) between the RA and Control groups in at least 19 hallmark gene sets.

### CIBERSORTx immune infiltration (RA/Control)

**Figure 6:**
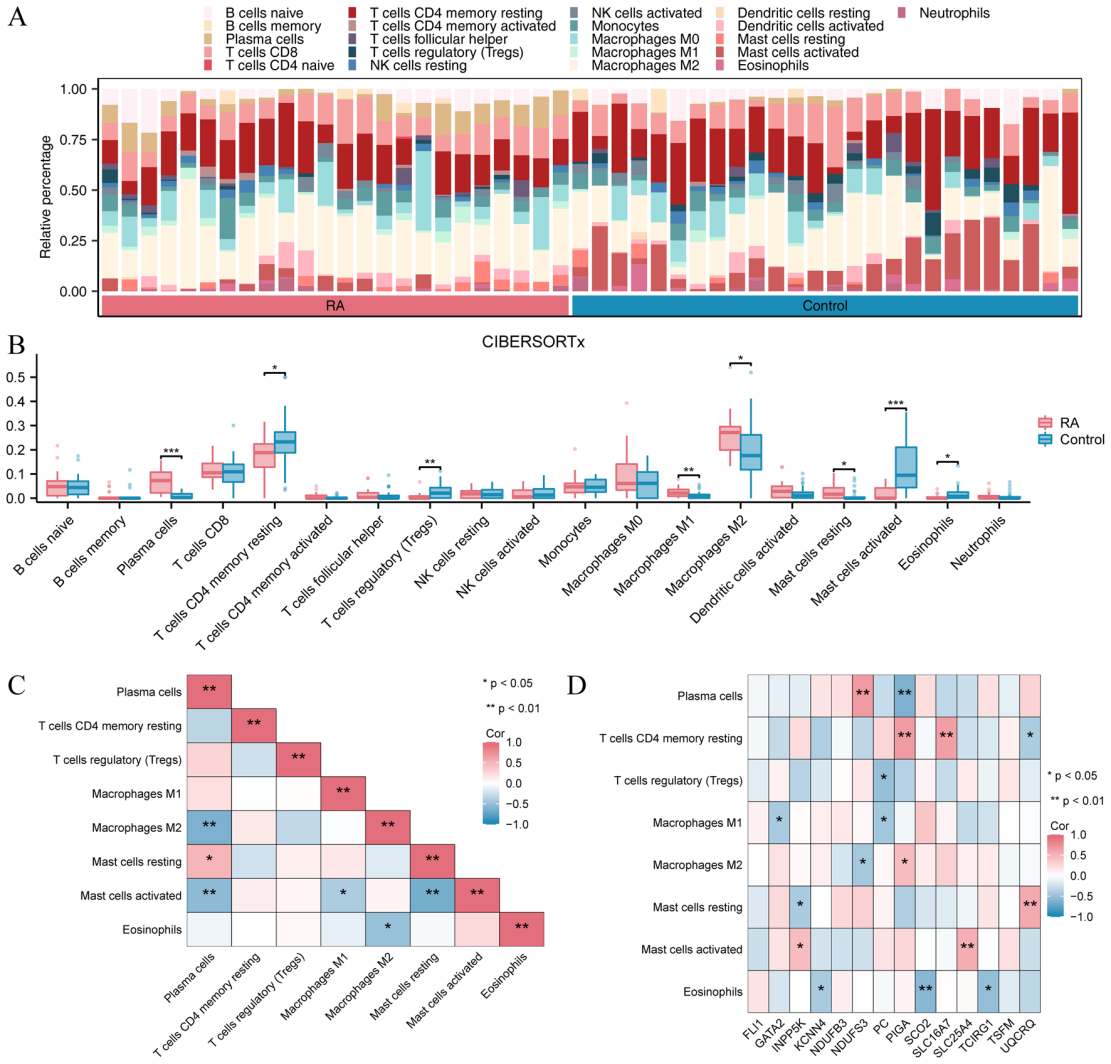
CIBERSORTx analysis to compare immune infiltration between the RA and Control groups. (**A**) Stacked histogram show the infiltration abundance of various immune cells in the RA dataset as calculated by the CIBERSORTx algorithm. Each sample is represented by different coloured stacked bars indicating the relative proportions of 22 different immune cells. (**B**) Box plots represent comparisons between the RA group and the Control group in terms of the abundance of different immune cell infiltrates. Each point represents a sample, and the box plots contain medians, quartiles, and show statistical significance by asterisks. (**C**) The heatmap showing the correlation between the eight immune cell infiltrates that were significantly different in the RA group versus the Control group. Like Graph A, colors and asterisks indicate correlation coefficients and significance. (**D**) The heatmap shows the correlation between specific immune cells and 14 key genes. As before, colours and asterisks indicate the degree and significance of the correlation. Statistical significance is indicated by asterisks in the group comparison graph (B) and the correlation heat map (CD). No asterisk represents *P* ≥ 0.05, indicating no statistical significance. An asterisk symbol (*) represents *P* < 0.05, indicating statistical significance. The symbol (**) represents *P* < 0.01, indicating high statistical significance. The symbol (***) represents *P* < 0.001, indicating high statistical significance. RA, rheumatoid arthritis.

We employed the CIBERSORTx algorithm to assess the abundance of 22 different immune cell types in the RA dataset sample to investigate the variation in immune infiltration between the RA and Control groups in the RA dataset. The histogram illustrates the distribution of immune cell infiltration abundance in the sample using the CIBERSORTx algorithm (Fig. 6A). Next, we created a comparative chart illustrating the variance in immune infiltration between the RA and Control groups in the RA dataset (Fig. 6B). The findings indicated that eight distinct types of immune cells (Plasma cells, resting memory CD4 T cell, T cells regulatory (Tregs), Macrophages M1, Macrophages M2, Mast cells resting, Mast cells activated, Eosinophils, Macrophages M0, Mast cells activated, Neutrophils) had statistically significant variances (*P* < 0.05). The heat map (Fig. 6C) illustrating the correlation between the infiltration levels of eight types of immune cells and 14 key genes. Additionally, the correlation heat map (Fig. 6D) demonstrated a significant positive linear correlation between gene UQCRQ and activated Mast cells and between gene SLC25A4 and mast cells resting (*r* > 0, *P* < 0.05).

### ssGSEA immune infiltration (RA/Control)

**Figure 7:**
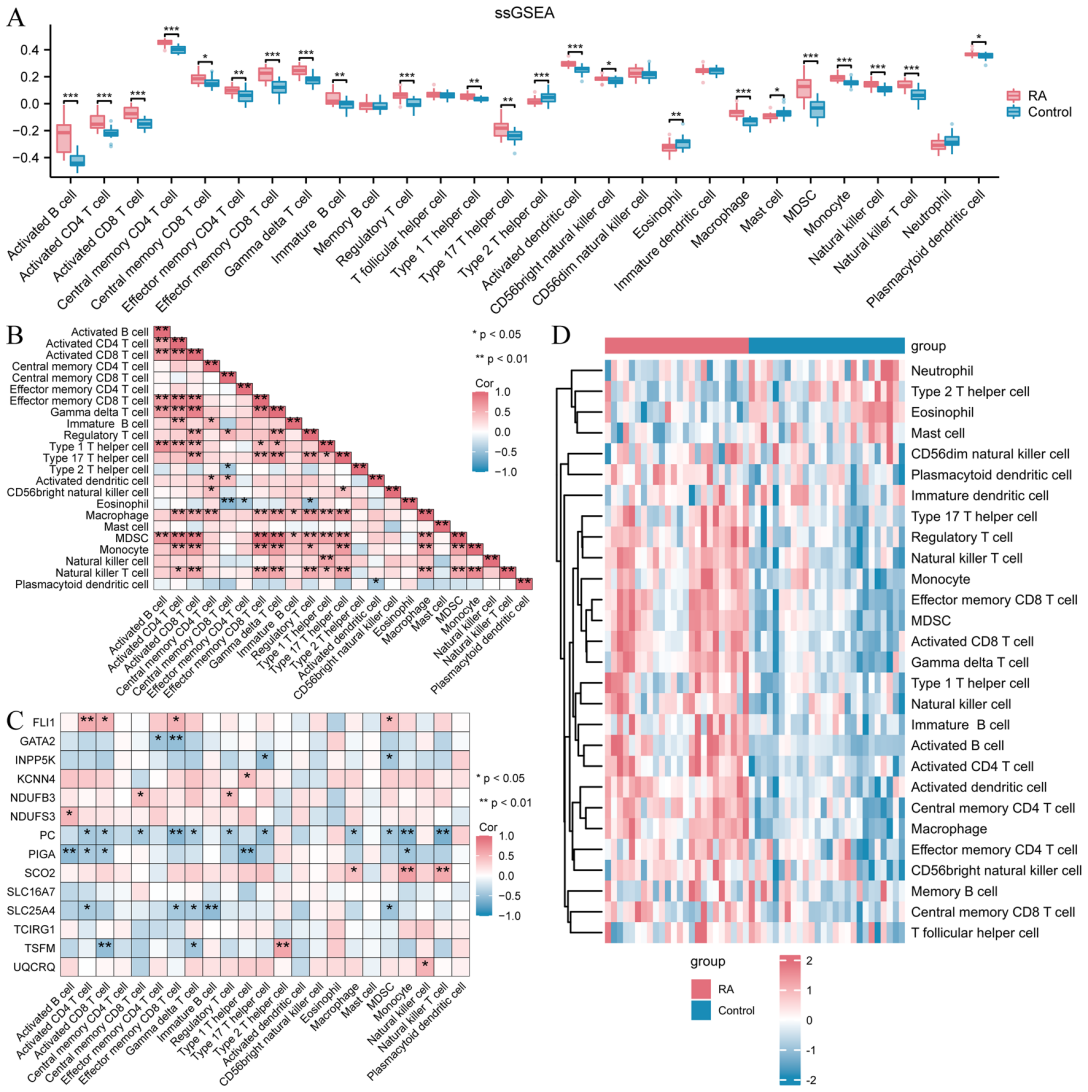
A comparison of immune infiltration between the RA and Control groups. (**A**) Box plots of ssGSEA analysis results. The horizontal axis lists the multiple immune cell types and the vertical axis indicates their enrichment fraction in the sample. Red represents the RA group and blue represents the control group. (**B**) Lower triangular heatmap of correlation between immune cell types obtained by ssGSEA analysis. Each box represents the value of the correlation coefficient between the two cell types, varying from − 1 (perfectly negative relationship, dark red) to 1 (perfectly positive relationship, dark pink), with 0 indicating no correlation. (**C**) As shown in Fig. 7B, a heat map demonstrating the correlation between immune cell types and a set of key genes. The key genes here such as FLI1 and GATA2 may play an important role in RA pathology. Again, the colours and asterisks represent correlation strength and statistical significance. (**D**) The immune cell infiltration of all samples between the RA group and the control group is shown as a heat map. The horizontal axis is the sample and the vertical axis is the immune cell type. The colour shades represent the fraction of different immune cell types enriched in each sample, with dark red representing a high enrichment fraction and light colours representing a low enrichment fraction. A significant difference in the infiltration of certain immune cell types can be observed between patients in the RA group and the control group. The asterisks in the comparison chart for groups (**A**) and the heat map for correlation (**B**, **C**) indicate statistical significance. A lack of asterisk indicates a *P*-value greater than or equal to 0.05, indicating no statistical significance. An asterisk (*) indicates a *P*-value less than 0.05, indicating statistical significance. The symbol (**) represents a *P*-value less than 0.01, indicating high statistical significance. The symbol (***) represents a *P*-value less than 0.001, indicating statistically significant results. RA, rheumatoid arthritis; ssGSEA, single-sample gene set enrichment analysis.

We employed the ssGSEA algorithm to compute the abundance of 28 distinct immune cell types present in the sample from the RA dataset to determine the variance in immune infiltration between the RA and Control groups within the RA dataset. The outcomes indicate that there is a significant disparity in infiltration abundance between the RA and Control groups (Fig. 7A) (*P* < 0.05) for 23 immune cell types. Next, we generated a heat map that illustrated the correlation between the abundance of immune cells and statistical significance in infiltration (Fig. 7B). Additionally, we created a correlation heat map to examine the relationship between these immune cells and 14 crucial genes (Fig. 7C). The findings indicated a notable favorable linear association between these immune cells (*r* > 0) and a significant positive linear correlation (*r* > 0) between genes (PC, PIGA, and SLC25A4) and these immune cells. In conclusion, a detailed heat map illustrating these immune cells' infiltration levels was created to compare the RA and Control groups in the RA dataset (Fig. 7D).

### Constructing LMRGs score

We determined RA based on the expression of 14 crucial genes in the dataset using the ssGSEA algorithm. We categorized the RA group into high and low groups using the median LMRGs score as the boundary. The ROC curve was used to examine the diagnostic impact of 14 crucial gene expressions on the High and Low groups (supplementary Fig. S3A–N). Graphs reveal that the genes KCNN4 (supplementary Fig. S3D, AUC = 0.819), SCO2 (supplementary Fig. S3I, AUC = 0.743), TCIRG1 (supplementary Fig. S3L, AUC = 0.757), and UQCRQ (supplementary Fig. S3N, AUC = 0.743) exhibit a certain level of diagnostic effectiveness on the High and the Low groups.

### CIBERSORTx immune infiltration (High/Low)

We employed the CIBERSORTx algorithm to determine the abundance of 22 different immune cells in the RA sample and examine the variation in immune infiltration between the High and Low groups in the RA dataset. Initially, a stacked histogram was employed to display the presence of immune cells in the sample using the CIBERSORTx algorithm (Supplementary Fig. S4A). Next, we generated a correlation heatmap for the immune cells and 14 crucial genes by plotting them together. The figure illustrates a clear positive linear relationship between CD8 T cells and gene UQCRQ, as well as activated NK cells and genes (GATA2 and TSFM) in the High and Low groups (*r* > 0, *p* < 0.05).

### ssGSEA immune infiltration (High/Low)

We employed the ssGSEA algorithm to compute the abundance of 28 different immune cells in the samples from the RA dataset to examine the variance in immune infiltration between the High and Low groups in the RA dataset. The findings indicate that the PC gene and immune cells in the samples from the High group predominantly exhibit a negative linear correlation (*r* < 0), while the SCO2 gene and immune cells in the samples from the Low group primarily exhibit a linear correlation (*r* > 0). The findings indicated that the group with a high LMRG score had increased immune cell infiltration abundance, whereas the group with a low LMRG score displayed decreased infiltration abundance(Supplementary Fig. S5C).

### Consistency clustering to construct RA disease subtypes

We analyzed the differences of 14 key gene expressions in the RA dataset of RA patients using the R package 'ConsensusClusterPlus.' We identified distinct RA-related disease subtypes by consensus clustering and ultimately classified them into two groups: cluster1 and cluster2 (Supplementary Fig. S6A). RA disease subtype 1 (cluster1) has 50 samples, while RA disease subtype 2 (cluster2) has 38. Additionally, we presented the CDF plot for the cumulative distribution function of the consistent cluster in the findings (Supplementary Fig. S6B), along with various clusters. Supplementary Fig. S6C presents the delta plot of the area beneath the cumulative distribution function curve for the number of categories. The comparison diagram of the 14 essential genes in the RA-dataset between cluster1 and cluster2 (Supplementary Fig. S6D) revealed statistically significant variations in genes (GATA2, KCNN4, PIGA, SLC16A7, TCIRG1, and UQCRQ) between cluster1 and cluster2 (*P* < 0.05). Additionally, the PCA plot for the RA group in the RA dataset (supplementary Fig. S6E) revealed an improved clustering effect that remained consistent. Next, the ROC curve indicated that the GATA2, KCNN4, NDUFS3, PIGA, TCIRG1, and UQCRQ genes positively impacted cluster1, while cluster2 exhibited enhanced predictive accuracy(Supplementary Fig. S6F–J).

### CIBERSORTx immune infiltration (cluster1/cluster2)

We employed the CIBERSORTx algorithm to compute the abundance of 22 distinct immune cell types in the RA sample to examine the variance in immune infiltration between cluster1 and cluster2 groups in the RA dataset. The histogram illustrates the distribution of immune cell infiltration abundance in the sample using the CIBERSORTx algorithm Supplementary Fig. S7A). The findings indicated that four types of immune cells (CD8 T cells, CD4 T cells in a resting memory state, resting NK cells, and resting mast cells) exhibited a statistically significant disparity (Supplementary Fig. S7B) (*P* < 0.05). Next, the correlation heatmap showed that in the cluster1 group samples.

### ssGSEA immune infiltration (cluster1/cluster2)

We determined the variations in immune infiltration between cluster1 and cluster2 groups using the ssGSEA algorithm (Supplementary Fig. S8A, B). A comprehensive heat map illustrating the infiltration abundance of these immune cells in the RA dataset (Supplementary Fig. S8C). The findings indicated a high number of immune cells in the cluster1 group. The cluster2 group has a lower infiltration abundance than the prevailing trend.

### The network of PPI and networks predicting mRNA-miRNA, mRNA-TF, mRNA-drug network, and protein domains

**Figure 8:**
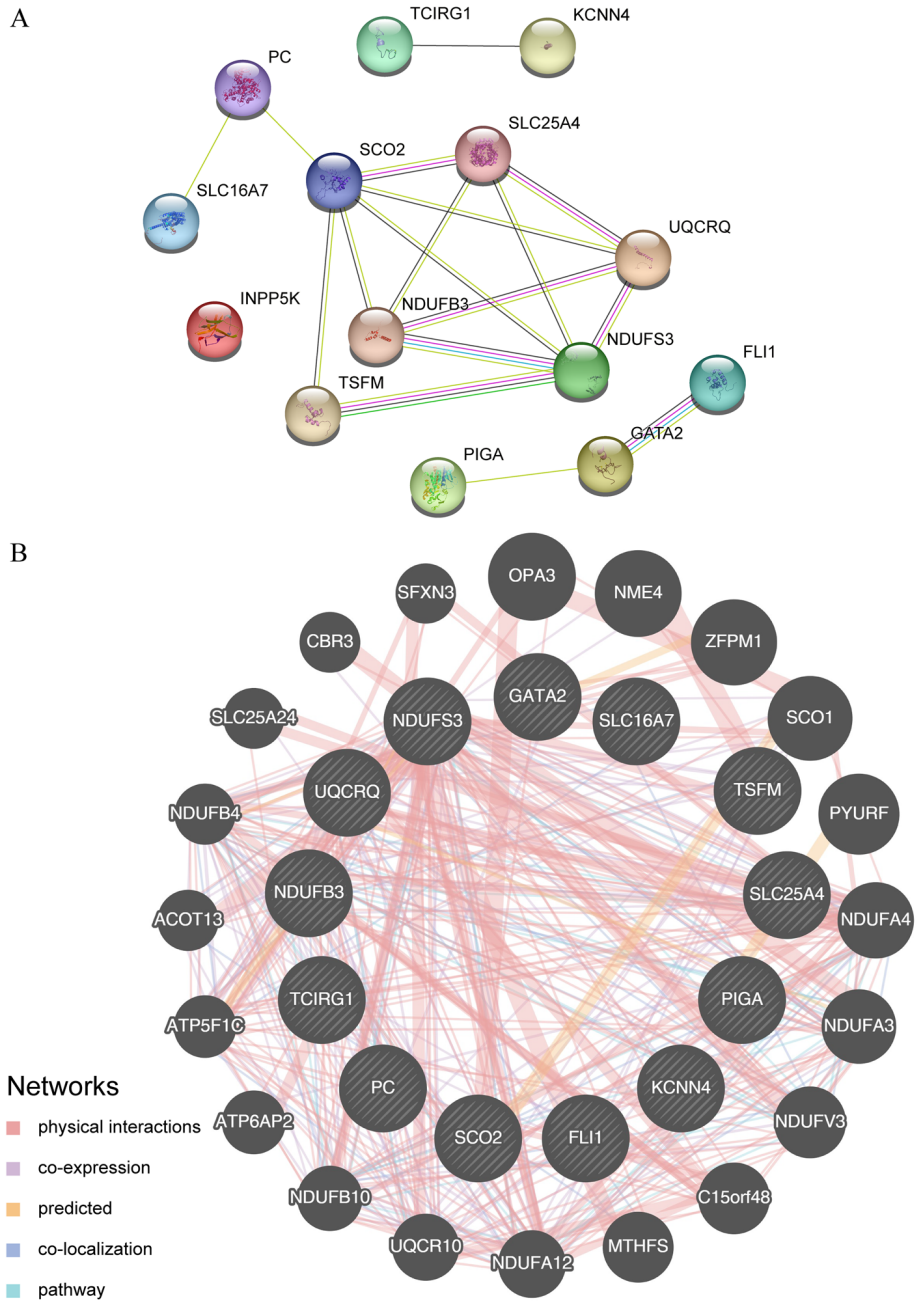
PPI interaction network. (**A**) Network of essential genes. The GeneMANIA website of (**B**) key genes predicts the network of interactions among genes with similar functions. A’s inter-structured network is gathered and exported from the STRING database, with a minimum interaction score of 0.150. The Gene MANIA website collects and exports the interconnected network structure of (**B**) black circles with white slashes represent the input key genes, while black circles represent predicted functionally similar genes without white slashes. Red lines indicate physical interactions between genes, purple connections represent co-expression relationships, yellow connections represent predicted connections, purple connections represent co-localization relationships between genes, and sky blue lines represent pathway-related relationships between genes. PPI, protein–protein interaction.

**Figure 9:**
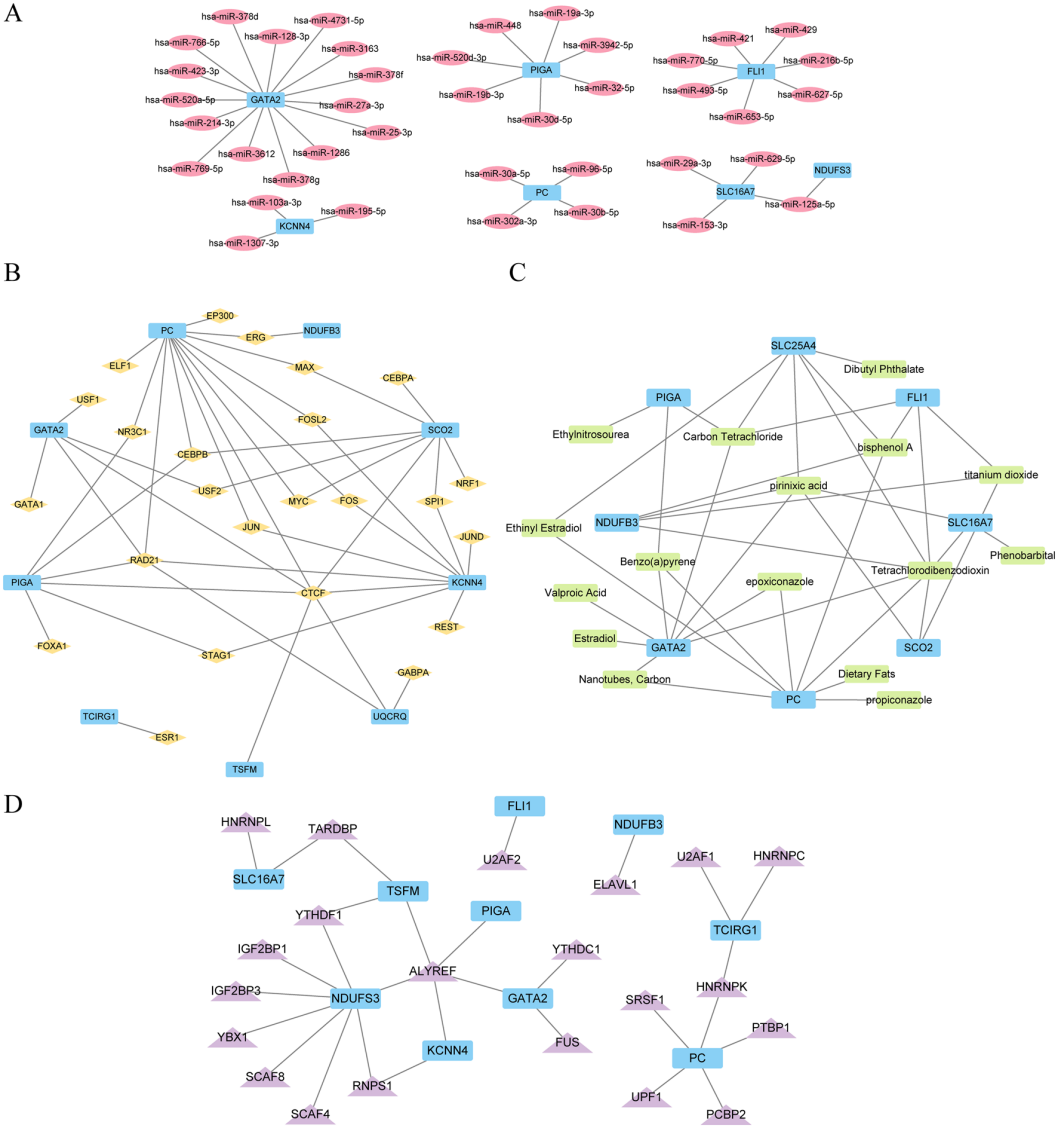
miRNA, TF, drug, RBP prediction network of key genes. (**A**) The network for predicting mRNA-miRNA interactions of important genes. Blue rectangles represent the mRNA, while red ovals represent miRNAs in the prediction network. The interaction data is sourced from the ENCORI database. (**B**) mRNA-TF prediction network for key genes. The blue rectangles symbolize mRNA, while the yellow diamonds symbolize TFs in the prediction network. The interaction data is sourced from the ChIPBase 3.0 database. (**C**) mRNA-drug prediction network for key genes. The blue rectangle represents mRNA, while the green rectangle represents the drug in the prediction network. The interaction data is sourced from the (**D**) Gidb database. Network prediction of hub genes for mRNA-RBP. The blue rectangles depict mRNA, while the purple triangles depict RBPs in the prediction network. The interaction data is sourced from the ENCORI database. Transcription factor (TF) is a protein that binds to RNA (RNA binding protein, RBP).

We examined the PPI of 14 crucial genes using the STRING database. A PPI interaction network of 13 key genes (excluding gene INPP5K) was obtained with a minimum confidence parameter (required interaction score) set at 0.150, indicating that the minimum required interaction score was 0.150 (Fig. 8A). Furthermore, we utilized the GeneMANIA website (Fig. 8B) to anticipate and build the interaction network of the functionally analogous genes associated with these 13 pivotal genes. This allowed us to examine their physical interaction relationship, co-expression, prediction, co-localization, pathway connection, and other related factors information (Fig. 9).

The ENCORI database was used to analyze mRNA-miRNA data and predict miRNAs interacting with important genes. mRNA-TF data were analyzed using the ChIPBase3.0 database and TFs interacting with key genes were identified. Cytoscape software was used to visualize the mRNA-miRNA interaction network (Fig. 8A), and the mRNA-TF interaction network (Fig. 8B). Supplementary Table S7 describes in detail the interactions between mRNAs and miRNAs as well as specific mRNA-TF interactions.

We predicted drugs interacting with important genes using mRNA-drug information from the DGidb database. And we visualized the mRNA-drug interaction network using Cytoscape software (Fig. 8C). The network contained eight mRNAs (SLC25A4, GATA2, PC, SCO2, SLC16A7, FLI1, NDUFB3, and PIGA) and 16 drugs. Supplementary Table S8 shows the interactions of specific mRNAs with drugs.

RBPs interacting with key genes were predicted using mRNA-RBP data from the ENCORI database. mRNA-RBP interaction networks were visualized using Cytoscape software and plotted in Fig. 8D. The interaction network consisted of 10 mRNAs (FLI1, GATA2, KCNN4, NDUFB3, NDUFS3, PC, PIGA, SLC16A7, TCIRG1, and TSFM) and 21 RBPs. Supplementary Table S9 lists specific mRNA-RBP interactions.

## Discussion

RA, a chronic autoimmune disorder, is primarily distinguished by inflammation of the synovium and damage to the joints. Research has confirmed that the swollen joints of RA patients are a site for a low-oxygen environment, leading to a disrupted lactate metabolism and lactate buildup. Lactate is currently acknowledged as a facilitator of combustion in RA, starting from the initial phases of inflammation and extending to the later stages of bone loss, rather than solely being considered a metabolic byproduct of glycolysis^6^. Several studies have indicated that lactate metabolism influences the regulation of inflammatory pathways and immune cell infiltration in autoimmune diseases^5,32^. For this research, we employed bioinformatics analysis and machine learning techniques to detect biomarkers associated with lactate metabolism in RA. We also explored the correlation between these biomarkers and immune cell infiltration and conducted preliminary investigations into their potential molecular pathways in the RA progression. We built an SVM model to screen the gene set. The key genes were analyzed using GO and KEGG analyses. CIBERSORTx and ssGSEA algorithms were utilized to perform GSEA, GSVA, and immune infiltration analyses. The STRING database was utilized to construct PPI networks.

LMRGDEGs were obtained by intersecting the differentially expressed genes identified through intergroup analysis between the RA and control groups with LMRGs. Among these 14 genes, the association with the highest positive linear correlation is between GATA2 and TCIRG1, followed by PIGA and SLC16A7, TCIRG1 and UQCRQ, and TCIRG1 and KCNN4. GATA2-AS1, transcribed by GATA2, was recently discovered to coordinate the activation of the glycolytic pathway dependent on HIF1 and the maintenance of mitochondrial biogenesis independent of HIF1^33^. Abnormal GATA2 expression and somatic mutations are linked to tumor promotion and inhibition^34^. KCNN4 regulates macrophage multinucleation in inflammatory conditions and bone homeostasis. Enhancement of cell metabolism by KCNN4 contributes to the malignant progression of HCCs^35^. SLC16A7 is a monocarboxylate transporter in the 14-gene SLC16 gene family. L-lactate, pyruvate, and ketone bodies are moved across the plasma membrane by linking them with protons. Besides, it plays a role in T-lymphocyte activation, intestinal metabolism, gluconeogenesis, drug transport, metabolic pathways, and the energy metabolism of skeletal muscle, cardiac muscle, and cancer cells^36^. These studies indicate that GATA2, KCNN4, and SLC16A7 might be involved in regulating lactate metabolism in RA. PIGA participates in phosphatidylinositol production on the endoplasmic reticulum membrane based on N-acetylglucosamine synthesis. Inherited metabolic disorders^37^ heavily rely on this reaction. The discovery of UQCRQ suggests that it could serve as a potential biomarker for predicting the response to abatacept/methotrexate in RA patients^38^. The TCIRG1 gene codes for the a3 subunit of the vacuolar ATPase proton pump, a significant variant. This variant plays a crucial role in the transportation of secretory lysosomes and the acidification of the resorption lacuna. Lack of TCIRG1 causes dysfunctional osteoclasts to ablate bone ineffectively^39,40^. Nevertheless, the existing proof fails to distinctly clarify the connection between RA and PIGA, UQCRQ, and TCIRG1; therefore, comprehensive research is necessary to shed light on this.

The primary cause of RA synovitis and joint damage is intricate interactions and the activation of immune cells that infiltrate the affected area^11,41^. Furthermore, this study identified LMRDEGs as being implicated in immune responses related to RA. Earlier research has discovered that suppressing KCNN4 via the control of Ca^2+^ communication diminishes the formation of multiple nuclei in macrophages and enhances bone density and the overall medical results in arthritis^42^. Macrophages within the synovial tissue potentially preserve balance and control inflammation in RA^43^. GATA2 is vital in differentiating dendritic cell (DCs) progenitors by regulating lineage-specific transcription factors determining the cell fate between myeloid and T-lymphocyte lineage^44^. According to a recent study, tumor-associated macrophages (TAMs) may regulate the heme oxygenase (HO-1) expression level by controlling SLC25A4, promoting M2 macrophage polarization, and enhancing tumor metastasis. Meantime, particular flaws in SLC25A4 trigger the activation of hypoxia-inducible factor (HIF-1α) within inflammatory macrophages, consequently fostering heightened lactate dehydrogenase (LDH) expression levels and concurrent elevation in glycolysis^45,46^. Furthermore, studies have demonstrated the crucial role of regulatory T cells, natural killer cells, and dendritic cells in RA progression^47,48^. Therefore, there is coherence between the present findings and prior ones. Afterward, the RA database was split into High and Low groups based on their LMRG scores. According to the CIBERSORTx and ssGSEA algorithm findings, immune cells exhibit greater infiltration abundance in the high LMRGs scores group than in the low infiltration abundance group. Lactate could have two opposing effects. Activated immune cells prefer lactate as their primary energy source. However, lactate accumulation in the tissue microenvironment acts as a signaling molecule that restricts the activity of immune cells^32^. Therefore, one could speculate that the distinct LMRDEG expressions in RA control the lactate metabolic pathways, leading to impaired immune cell function. However, the mechanism by which the lactate metabolic pathway influences the immune response to RA remains unclear. Further experimental investigations are necessary to examine how LMRDEGs involved in lactate metabolism regulate immune response in RA.

Further examination of variations in immune cell infiltration by LMRDEGS within RA databases. The findings indicated that the prevalence of immune cell infiltration differs between RA disease subcategories. This highlights the significance of LMRDEGs in the initial detection of RA. Subsequently, ROC analysis suggests that genes: GATA2, KCNN4, NDUFS3, PIGA, TCIRG1, and UQCRQ have valid diagnostic significance for RA. Despite the inability of previous research to pinpoint a precise mechanism for GATA2 in RA, it was discovered to function as a transcription factor that closely interacts with key genes in RA^49^. Substantially, GATA2 influences cell fate between the myeloid and T-lymphocyte lineage during DC development by regulating lineage-specific transcription factors in DC progenitors^44^. Combined with ROC analysis results, GATA2 in RA might affect immune mechanisms by regulating dendritic cell differentiation. The KCNN4 gene is functionally operational, being present in synovial fibroblasts associated with RA, and plays a role in controlling cell growth and the secretion of harmful and pro-inflammatory substances^50^. NDUFS3, a pro-oxidant component of electron transport chain (ETC) complex I, regulates nonopsonic phagocytosis of bacteria in macrophages^51^. Although the exact cause of NDUFS3 in RA remains uncertain, certain research has indicated its role in the progression of various conditions, including systemic lupus erythematosus (SLE) and lung adenocarcinoma (LUAD)^52,53^. The present investigation observed a notable rise in immune cell infiltration, specifically macrophage infiltration, in RA patients. As mentioned earlier, the findings remain unchanged. Significant associations between these crucial genes and RA were identified, suggesting their potential as biomarkers for RA.

The miRNAs that interact with crucial genes were predicted using the ENCORI database. Several of these 40 miRNAs have been identified as playing a role in the RA progression. The KCNN4 gene contains the following microRNAs: has-miR-103a-3p, hsa-miR-195-5p, and hsa-miR-1307-3p. According to certain research, patients diagnosed with established RA can identify elevated miR-103a levels in complete blood samples linked to the disease severity^54^. Elevated levels of miR-125a-5p are observed in RA patients, suggesting their role in the advancement and occurrence of the disease^55^. SLC16A7 is linked to miRNA has-miR-125a-5p. The network of mRNA-TF interactions reveals that 24 transcription factors are involved in RA. There is a positive correlation between FOS and nuclear factor interleukin 3 (NFIL3) in the peripheral blood of RA patients, as well as an abnormal inflammatory cytokine and inflammatory response linked to high NFIL3 expression^56^. RA-induced activation of the PI3K-AKT and mTOR signaling cascades could potentially enhance MYC expression in TEMRA CD8+ T cells, consequently modulating the glycolysis transcriptional pathway in RA^57^. The mRNA-drug interactions network lists 16 drugs that might have potential therapeutic effects in RA. Administering estradiol as a hormone treatment for managing RA during premenstrual exacerbations could yield positive outcomes^58^. Phenobarbital has been reported to inhibit the proliferation and viability of rabbit synoviocyte cell line HIG-82^59^. The mRNA-RBP interaction network revealed that 21 RBPs were linked to RA. RA involves the interaction between a long non-coding RNA (lncRNA) called ENST00000509194 and RNA-binding protein ELAVL1, playing a role in the migration and invasion of fibroblast-like synoviocytes (FLSs)^60^. Further investigation is required to examine the involvement of these crucial genes in RA despite the validation of certain predictions from different databases in previous research. This may offer a fresh outlook for additional experimental verification in the future.

Although we employed bioinformatics and machine learning techniques to identify potential biomarkers of RA in this study, we must acknowledge its limitations. And different analyses (CIBERSORTx, ssGSEA and LMRGscore) have sometimes produced conflicting results. We believe there are several reasons for this: (1) Methodological variability: different immune infiltration analysis methods may be based on different algorithms and assumptions, leading to differences in results. (2) Biological complexity: The immune system is a complex system with mutual regulation and interaction between immune cells. Therefore, under different analytical methods, it is possible to see results where different immune cells interact with each other, leading to differences in results. (3) Sample differences: possible sample heterogeneity and individual differences between the RA and Control groups may also contribute to differences in the observed immune infiltration results. The selection and handling of the study samples may have an impact on the results. From a long term perspective, investigating the mechanism of action of the lactate metabolic pathway involved in immune cell function will require studies conducted in vitro and in vivo. Moreover, this study lacked appropriate clinical correlation studies.

To summarize, this research offers initial recognition of possible markers linked to lactate metabolism in RA and insight into how it is connected to immune cells associated with RA. KCNN4 and SLC25A4 may regulate macrophage function during RA development via the lactate metabolic pathway. Additionally, GATA2 may participate in the lactate metabolic pathway to regulate the immune mechanism of DC cells involved in RA. These research findings present fresh perspectives on the diagnosis, lactate metabolic routes, and immune molecular mechanisms associated with RA.

### Supplementary Information

Supplementary Information.

## Data Availability

Datasets analyzed for this study (GSE1919, GSE29746 and GSE55235) are available from the GEO database.
