# Supplementary material for: Unveiling the link between lactate metabolism and rheumatoid arthritis through integration of bioinformatics and machine learning
Source: Sci Rep. 2024 Apr 22;14:9166. doi: 10.1038/s41598-024-59907-6 (PMC11033278; doi:10.1038/s41598-024-59907-6)
Supplement: Supplementary file 1 — Supplementary Information. [file 41598_2024_59907_MOESM1_ESM.pdf]

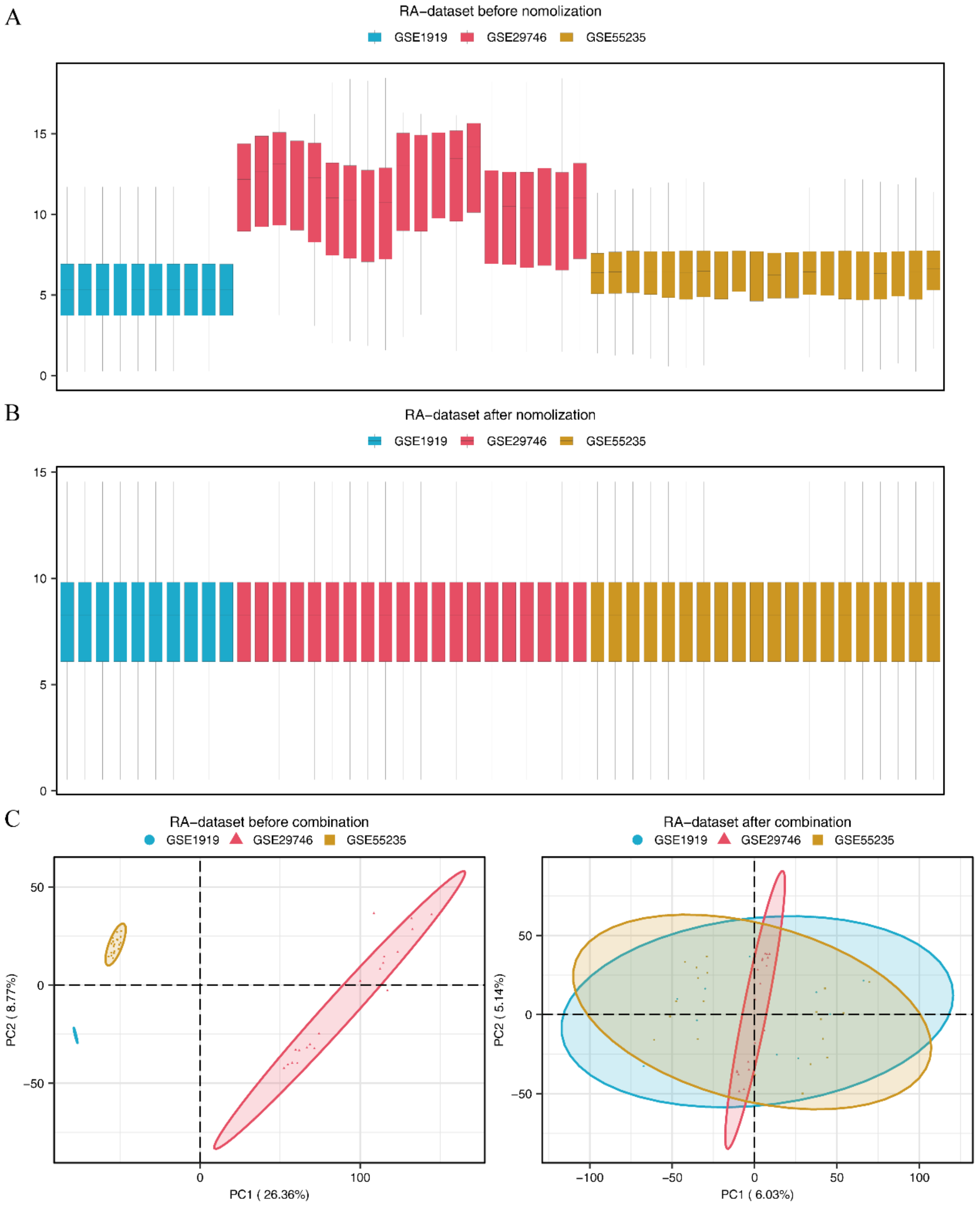

Figure S1. A boxplot and PCA plot for the RA data set.

AB. The boxplot of the sample distribution of the dataset RA-dataset before (A) and after (B) normalization. CD. The PCA plot of the data set RA-dataset before (C) and after (D) batching. RA, rheumatoid arthritis . PCA, principal component analysis.

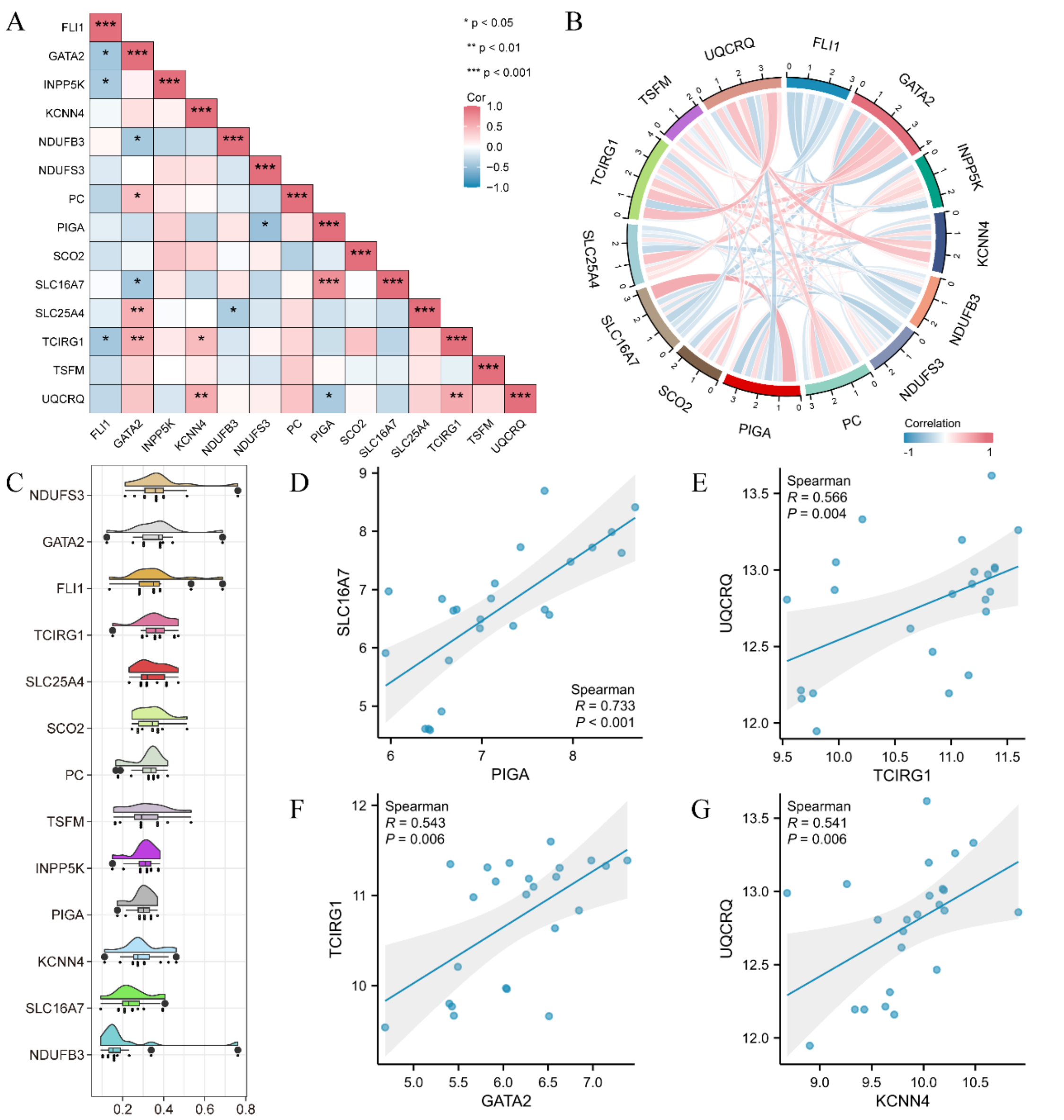

Figure S2. Analysis of the correlation and functional similarity of important genes

A-B. The RA dataset displays the correlation heatmap (A) Heatmap showing correlation analysis between genes (e.g. FLI1, GATA2, etc.). Box colours represent correlation coefficients; red indicates positive correlation, blue indicates negative correlation, and asterisks indicate the level of statistical significance. (B) Circular linkage plots showing correlations between genes and key genes. The coloured lines indicate the correlation between the variables, the closer to the centre of the line the stronger the correlation, and the colour represents the positive or negative correlation coefficient.

C. Violin plot showing the distribution of expression levels of key genes grouped using colours.

Each gene shows the distribution of its expression value, where the black dot represents the median. D-G. Scatter plots and trend lines show the correlation between specific key genes. In the scatter plot, the horizontal and vertical coordinates indicate the expression levels of different genes and the trend line indicates the correlation between these genes, while Spearman correlation coefficients and p-values are provided. The correlation heat map (A) uses an asterisk to indicate statistical significance. An absence of an asterisk (*) indicates that the value is equal to or greater than $P \geq 0.05$, meaning there is no statistical significance. On the other hand, a single asterisk (*) indicates that the value is less than $P < 0.05$, indicating statistical significance. Lastly, two asterisks (**) indicate that the value is less than $P < 0.01$, indicating high statistical significance. A correlation scatter plot has a strong correlation when the correlation coefficient (r) is above 0.8, a moderate correlation between 0.5–0.8, a weak correlation between 0.3–0.5, and below 0.3, it is either weak or irrelevant. RA: rheumatoid arthritis.

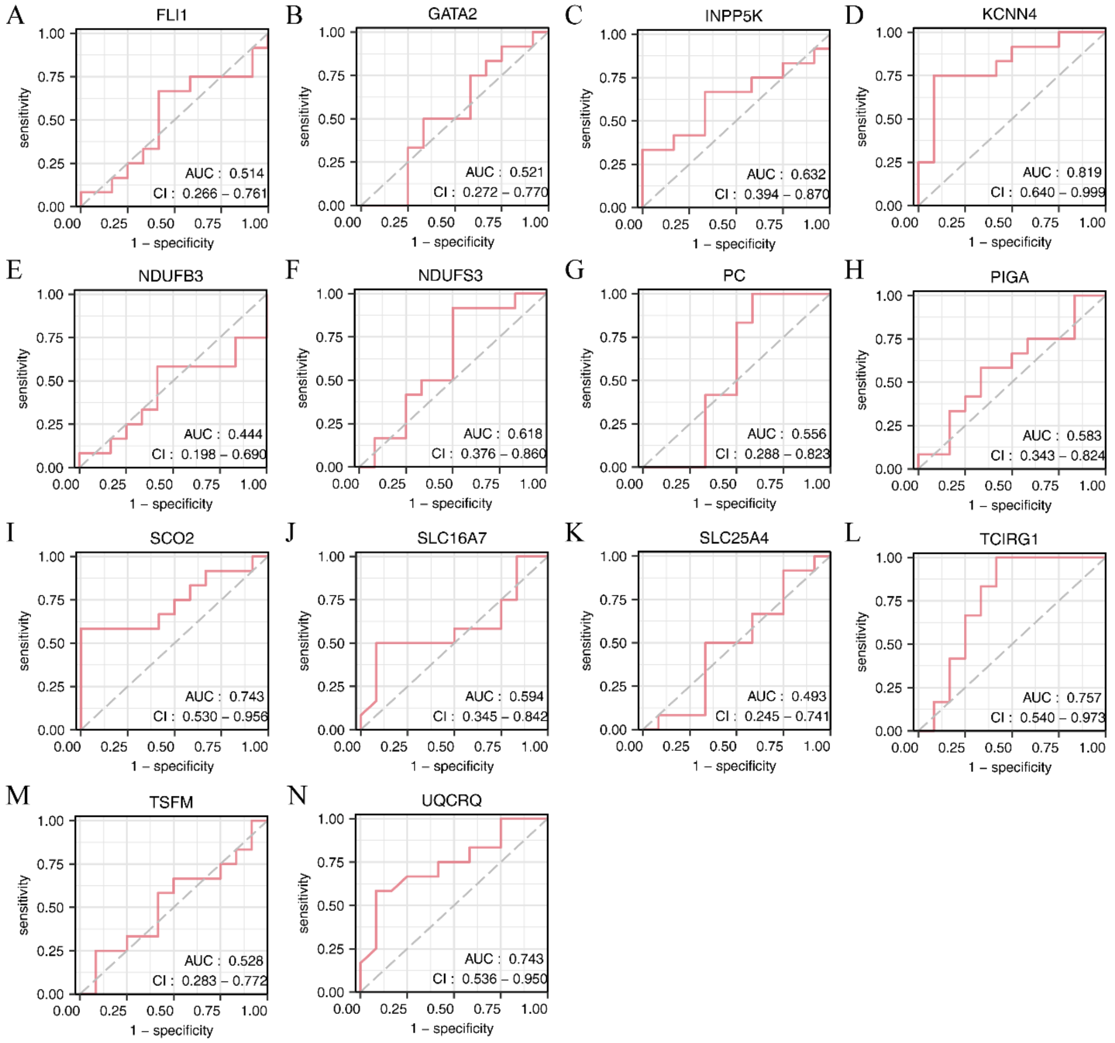

Figure S3. Construction of LMRGs score

The outcomes of the receiver operating characteristic (ROC) curve for A-N. The key genes are displayed in the RA-dataset LMRGs, with a scoring difference between the high and low groups. A. ROC curve for the FLI1 biomarker with an AUC of 0.514. B. ROC curve for the GATA2 biomarker with an AUC of 0.521. C. ROC curve for INPP5K biomarker with an AUC of 0.632. D. ROC curve for the KCNN4 biomarker with an AUC of 0.819. E. ROC curve for the NDUFB3 biomarker with an AUC of 0.444. F. ROC curve for the NDUFS3 biomarker with an AUC of 0.618. G. ROC curve for PC biomarkers with an AUC of 0.556. H. ROC curve for the PIGA biomarker with an AUC of 0.583. I. ROC curve for the SCO2 biomarker with an AUC of 0.743. J. ROC curve for SLC16A7

biomarker with an AUC of 0.594. K. ROC curve for SLC25A4 biomarker with an AUC of 0.493. L. ROC curve for the TCRG1 biomarker with an AUC of 0.757. M. ROC curve for the T5FM biomarker with an AUC of 0.528. N. ROC curve for the UQCRQ biomarker with an AUC of 0.743. LMRGs are genes associated with oxidative stress and autophagy; RA is linked to gestational diabetes mellitus. ROC: receiver operating characteristic curve; AUC: area under the curve; TPR: true positive rate; FPR: false positive rate; and CI: confidence interval.

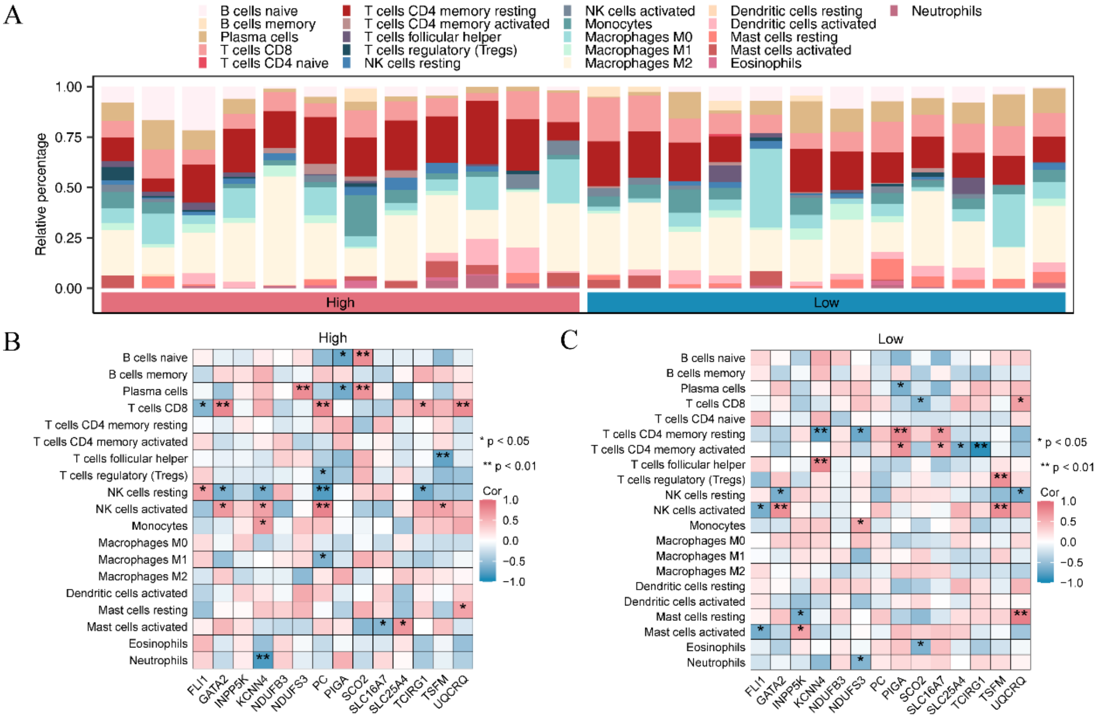

Figure S4. Comparison of immune infiltration analysis using CIBERSORTx between the High and Low groups

A. The CIBERSORTx algorithm analyzed the RA dataset and generated a stacked histogram presenting the abundance of immune cell infiltration in various samples. Each bar in the histogram represents a different type of immune cell, distinguished by its color. B. The CIBERSORTx algorithm generated a heat map illustrating the correlation between the presence of immune cells and important genes in the High-group samples of the RA dataset. The CIBERSORTx algorithm

generated a heat map illustrating the correlation between immune cell infiltration and important genes in the samples from the Low group in the RA dataset. The correlation heat map (BC) uses asterisks to indicate statistical significance. No asterisk indicates $P \geq 0.05$, meaning no statistical significance. The symbol * indicates $P < 0.05$, indicating statistical significance. The symbol ** indicates $P < 0.01$, indicating high statistical significance. RA: rheumatoid arthritis.

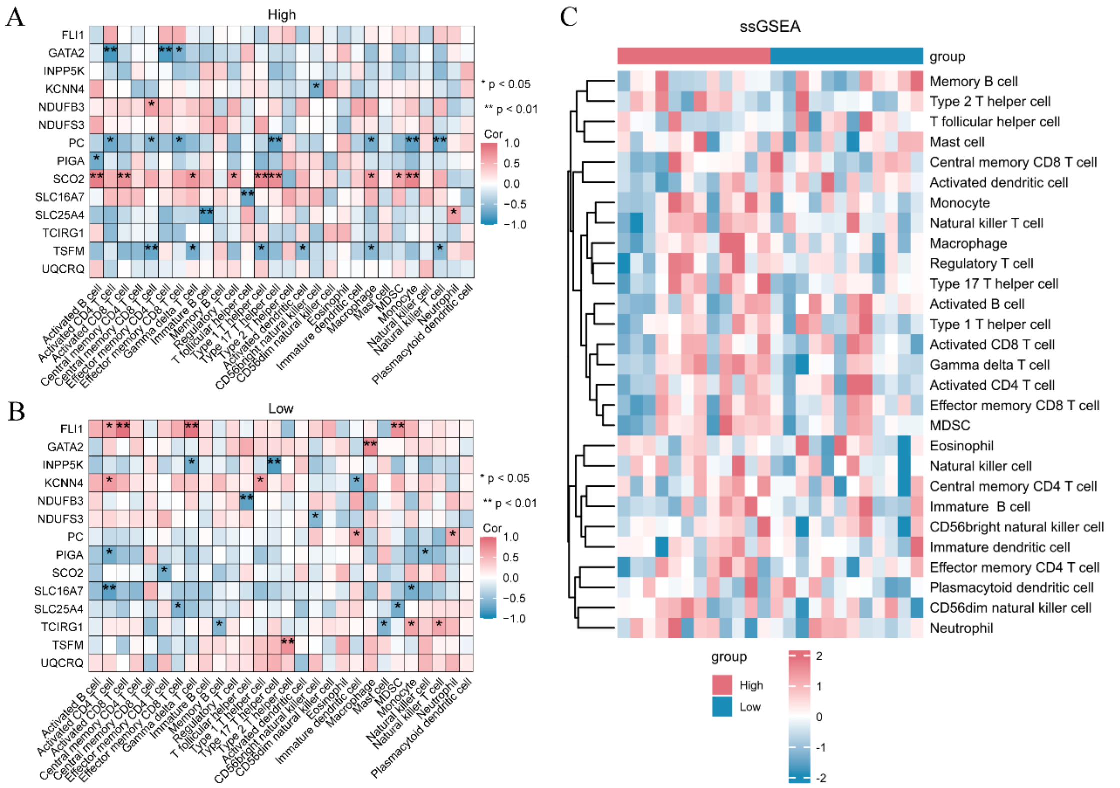

Figure S5. ssGSEA groups of High and Low exhibit varying levels of immune infiltration.

A. The ssGSEA algorithm generated a heat map illustrating the correlation between the presence of immune cells and important genes in the High-group samples of the RA dataset. B. The ssGSEA algorithm generated a heat map illustrating the correlation between the presence of immune cell infiltration and important genes in the Low group samples of the RA dataset. A detailed heat map displaying the abundance of immune cell infiltration between the High and Low groups in the RA dataset using the ssGSEA algorithm. The correlation heat map (A-B) uses asterisks to indicate

statistical significance. No asterisk indicates that $P \geq 0.05$, meaning there is no statistical significance. An asterisk (*) indicates that $P < 0.05$, which is statistically significant. Two asterisks (**) indicate that $P < 0.01$, indicating high statistical significance. RA: rheumatoid arthritis; ssGSEA: single-sample gene set enrichment analysis.

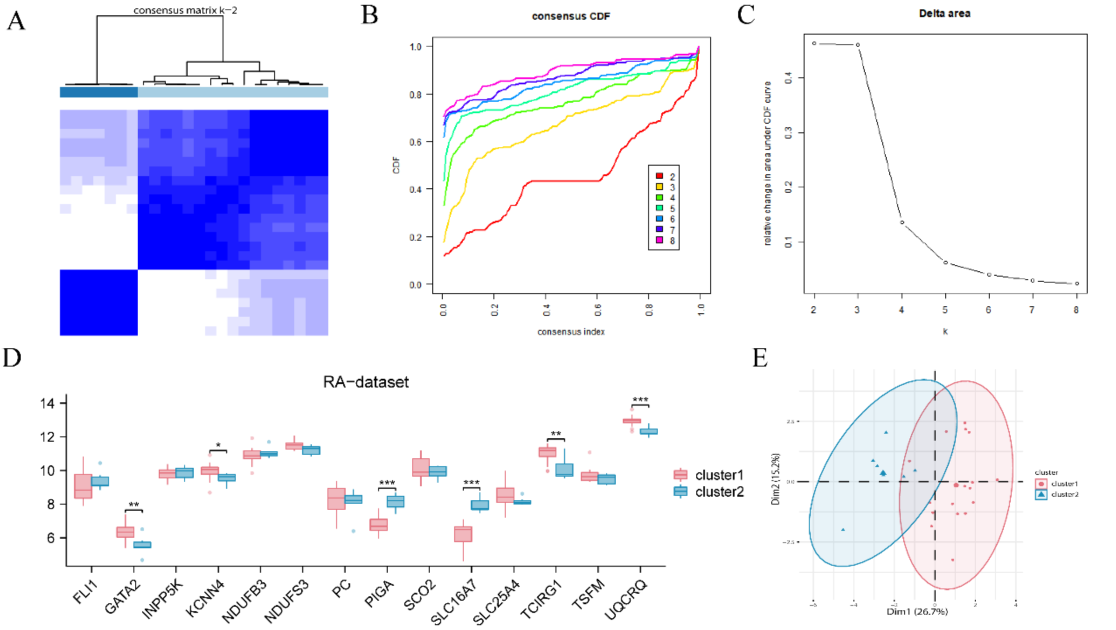

Figure S6. Consensus clustering to construct disease subtypes of RA

A. Consensus clustering (K = 2) results in a plot of RA disease. B-C graph (B) and Delta plot (C) area under the CDF curve for varying quantities of RA disease subcategories. D. The RA dataset presents the key genes determined from the group comparison graph results between disease subtype cluster1 and cluster2. E. The RA dataset displays a PCA graph based on clustering disease subtypes into cluster1 and cluster2.

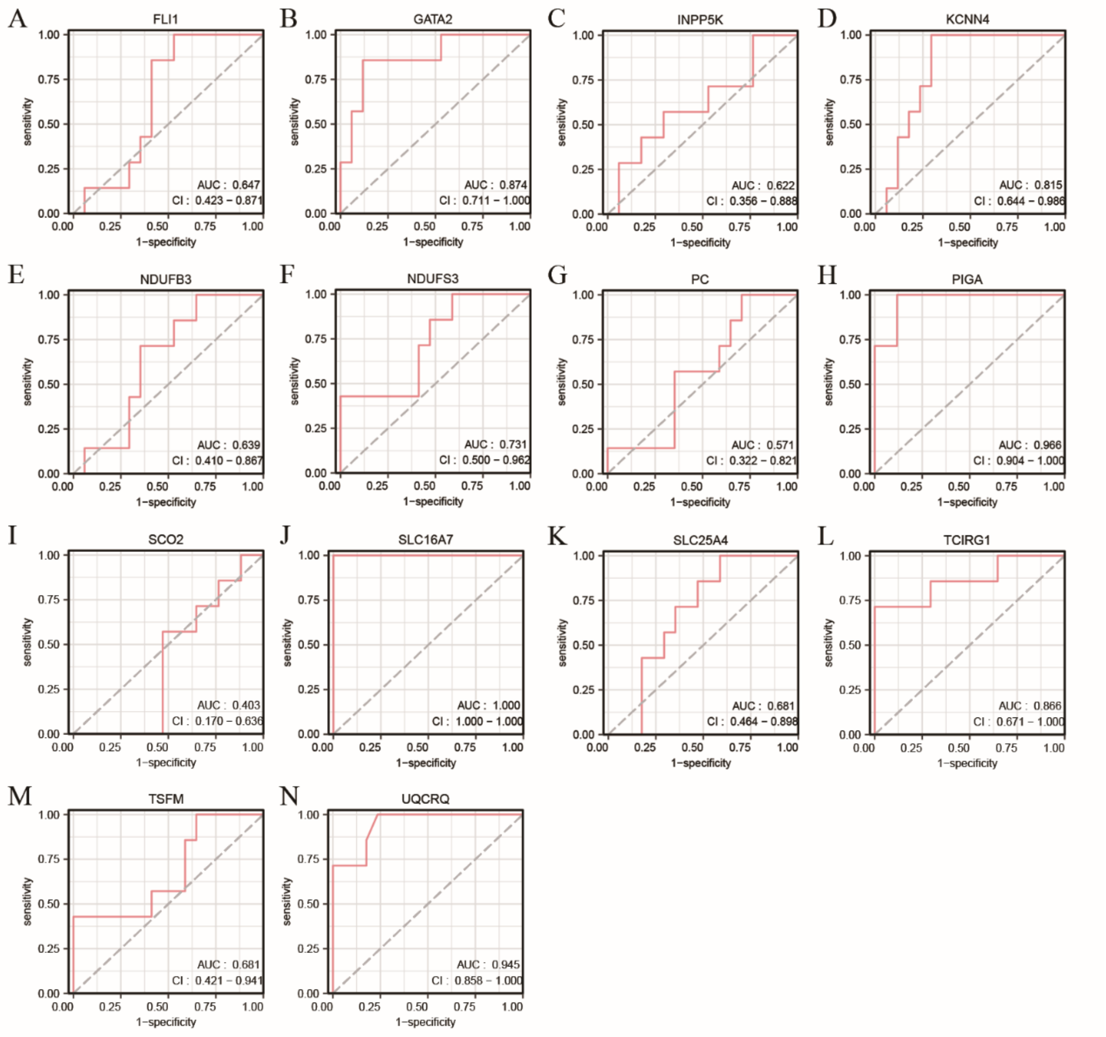

Figure S7. The key genes in disease subtypes of RA.

The RA dataset depicts the key genes based on the ROC curve results comparing the disease subtype cluster1 and cluster2. A. ROC curve for the FLI1 biomarker with an AUC of 0.647. B. ROC curve for the GATA2 biomarker with an AUC of 0.874. C. ROC curve for INPP5K biomarker with an AUC of 0.622. D. ROC curve for the KCNN4 biomarker with an AUC of 0.815. E. ROC curve for the NDUFB3 biomarker with an AUC of 0.639. F. ROC curve for the NDUFS3 biomarker with an AUC of 0.731. G. ROC curve for PC biomarkers with an AUC of 0.571. H. ROC curve for the PIGA biomarker with an AUC of 0.966. I. ROC curve for the SCO2 biomarker with an AUC of 0.403. J. ROC curve for SLC16A7 biomarker with an AUC of 1.000. K. ROC curve for SLC25A4 biomarker

with an AUC of 0.681. L. ROC curve for the TCRG1 biomarker with an AUC of 0.866. M. ROC curve for the T5FM biomarker with an AUC of 0.681. N. ROC curve for the UQCRQ biomarker with an AUC of 0.945. The accuracy of the AUC in the ROC curve (A–N) is low between 0.5–0.7, moderate between 0.7–0.9, and high above 0.9. RA refers to rheumatoid arthritis, CDF: cumulative distribution function; PCA: principal component analysis; ROC: receiver operating characteristic curve; AUC: area under the curve; TPR: true positive rate; FPR: false positive rate; and CI: confidence interval.

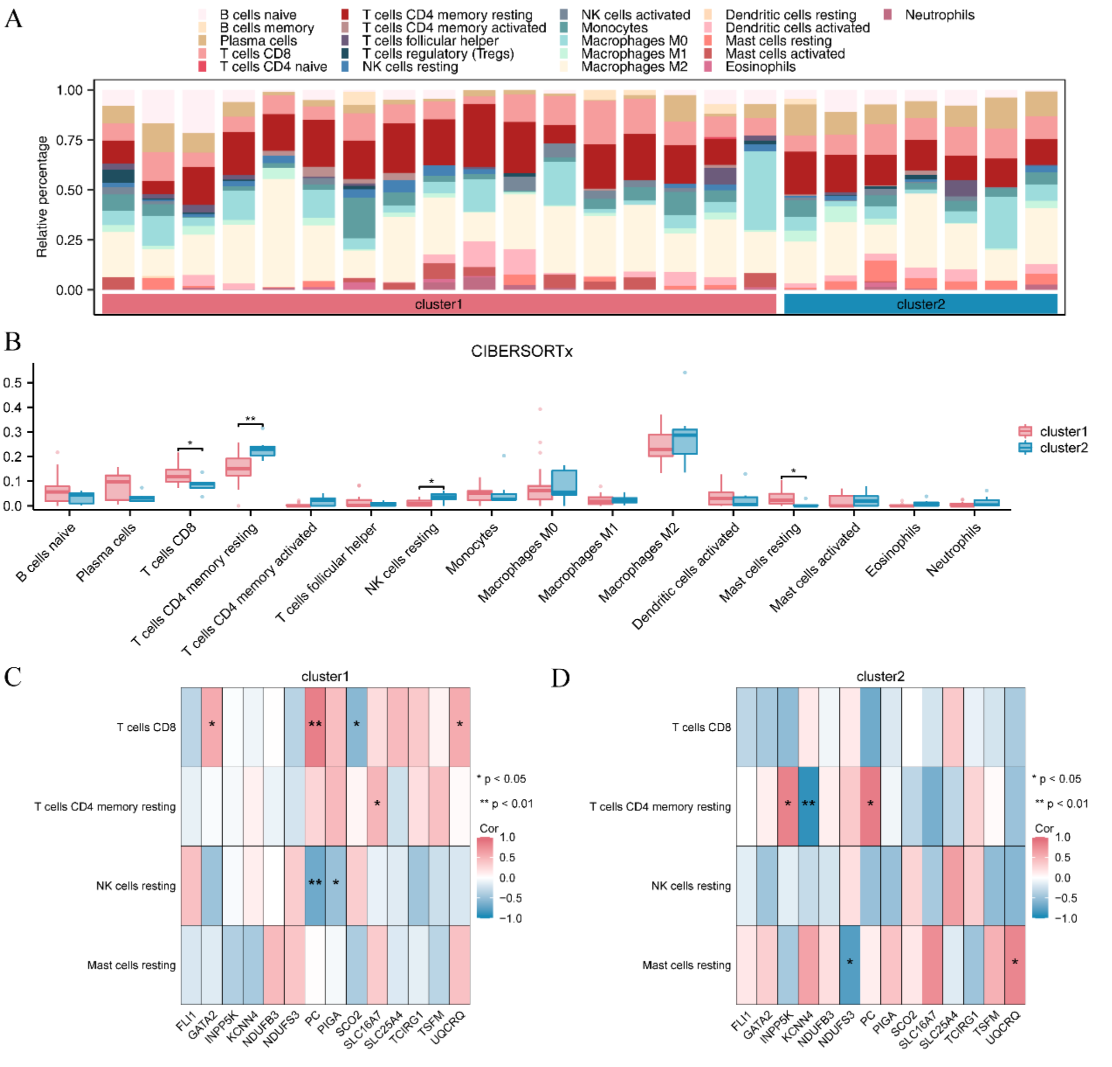

Figure S8. CIBERSORTx analysis to evaluate immune infiltration between the cluster1 and cluster2 groups.

A. The CIBERSORTx algorithm created a stacked histogram illustrating the distribution of immune cells in various samples of the RA dataset. Each bar in the histogram corresponds to a specific immune cell and is colored differently. B. The comparison diagram depicts the difference in immune cell infiltration abundance between the cluster1 and cluster2 groups in the RA dataset using the CIBERSORTx algorithm. The CIBERSORTx algorithm generated a heat map illustrating the correlation between the abundance of immune cell infiltration and key genes in the cluster1 group samples within the RA dataset. The CIBERSORTx algorithm generated a heat map illustrating the correlation between the abundance of immune cell infiltration and key genes in the cluster2 group samples within the RA dataset. The asterisks in the group comparison graph (B) and the correlation heat map (C-D) indicate the level of statistical significance. No asterisk indicates a *P* value greater than or equal to 0.05, meaning no statistical significance. One asterisk (*) indicates a *P*-value less than 0.05, indicating statistical significance. Two asterisks (**) indicate a *P*-value less than 0.01, indicating high statistical significance. Three asterisks (***) indicate a *P*-value less than 0.001 and high statistical significance. RA: rheumatoid arthritis.

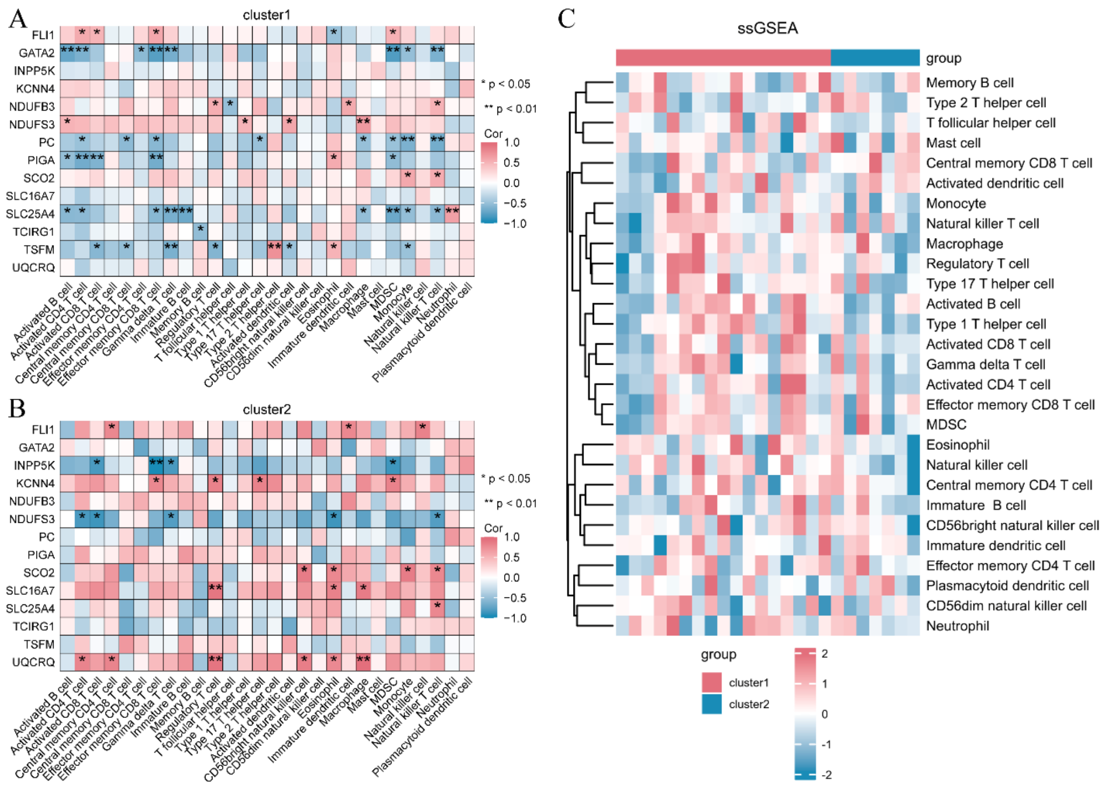

Figure S9. ssGSEA immune infiltration between cluster1 group and cluster2 group

A. The ssGSEA algorithm generated a heat map illustrating the correlation between the abundance of immune cell infiltration and key genes in the cluster1 group samples within the RA dataset. B. The ssGSEA algorithm generated a heat map illustrating the correlation between the abundance of immune cell infiltration and key genes in the cluster2 group samples within the RA dataset. The intricate heat map displays the abundance of immune cell infiltration in the cluster1 and cluster2 groups within the RA dataset using the ssGSEA algorithm. The correlation heat map (A-B) uses asterisks to indicate statistical significance. No asterisk indicates that $P \geq 0.05$, presenting no statistical significance. An asterisk (*) indicates that $P < 0.05$, displaying statistical significance. Two asterisks (**) indicate that $P < 0.01$, indicating high statistical significance. RA: rheumatoid arthritis; ssGSEA: single-sample gene set enrichment analysis.

Table S1. List of gene symbols of LMRGs.

LMRGs
LDHB
LDHA
EMB
SLC16A1
SLC16A3
SLC16A7
SLC16A8
SLC5A12
SLC5A8
LDHAL6A
LDHAL6B
LDHC
LDHD
COG8
COX16
CYP27A1
DNM1L
HPDL
HSD17B10
KARS1
MT-CO1
MT-CO2
MT-CO3
MT-ND1
MT-ND4
MT-ND5
MT-ND6
MT-TF
MT-TH
MT-TL1
MT-TQ
MT-TS2
MT-TW
MTRFR
NDUFAF3
NDUFB8
NDUFS2
SCO2
SDHB
SQOR
SUCLG1
SURF1

ACAD9
ACADM
AKR1D1
B3GALNT2
B4GAT1
CALR
CD46
CFH
CFI
CHEK2
COL4A1
CPT2
CRPPA
DAG1
FKRP
FKTN
FLI1
GAA
GATA1
GATA2
HBB
HELLPAR
HLA-DRB1
HMOX1
INPP5K
IRAK1
JAK2
KCNN4
KY
LARGE1
LIPA
LPIN1
LYST
MPL
MVK
MYC
OCRL
PIEZO1
PIGA
PITRM1
PLA2G6
PLEC
PNPLA2
POMGNT1

POMGNT2
POMK
POMT1
POMT2
RB1
RHAG
RHCE
RHD
RPS14
RXYLT1
SIL1
SLC19A1
SLC25A13
SLC4A1
SLC7A7
SPP1
STAT4
TCIRG1
TET2
TP53
USB1
VPS13A
ZNFX1
GFM1
MRPL3
NDUFB10
NDUFS4
AIFM1
ALDH4A1
COA8
COQ8A
COX10
COX14
COX15
COX20
COX4I1
COX6B1
COX8A
DARS2
ECHS1
FOXRED1
GFM2
HTRA2
LIPT1

LONP1
LRPPRC
MDH2
MECP2
MPV17
MRPS34
MT-ATP6
MT-ND2
MT-ND3
MT-TK
MT-TN
MT-TV
MTFMT
NARS2
NAXE
NDUFA1
NDUFA10
NDUFA11
NDUFA12
NDUFA13
NDUFA2
NDUFA4
NDUFA6
NDUFA8
NDUFA9
NDUFAF1
NDUFAF2
NDUFAF4
NDUFAF5
NDUFAF6
NDUFAF8
NDUFB11
NDUFB3
NDUFB9
NDUFS1
NDUFS3
NDUFS6
NDUFS7
NDUFS8
NDUFV1
NDUFV2
NUBPL
PDHA1
PDHX

PET100
PET117
PNPT1
RARS2
RMND1
SDHA
SLC13A3
SLC19A3
SLC25A19
SLC25A4
SLC39A8
TACO1
TIMMDC1
TMEM126B
TRAPPC12
TRMT10C
TXN2
AARS2
ACAT1
ACAT2
ADAMTS13
AGK
ATAD3A
ATPAF2
BCS1L
C1QBP
CA5A
CARS2
CHCHD10
CLPB
COQ2
COQ4
COQ9
COX5A
COX6A2
CYC1
DGUOK
DLD
DNAJC19
EARS2
FARS2
FASTKD2
FBXL4
FDX2

GOT2
GTPBP3
GYS2
HIBCH
HMGCL
HS6ST2
ISCA1
ISCU
LIAS
LIPT2
LYRM7
MICOS13
MIPEP
MPC1
MRPL12
MRPL44
MRPS14
MRPS16
MRPS22
MRPS28
MT-TI
MT-TL2
MT-TP
MTO1
NDUFC2
NFS1
NGLY1
OGDH
PC
PDP1
PDSS1
PDSS2
PHKG2
PMPCB
PNPLA8
PNPO
POLG
POLG2
PUS1
PYGL
RARS1
RNASEH1
RRM2B
SCO1

SERAC1
SFXN4
SLC25A10
SLC25A26
SLC25A3
SLC25A42
SOD1
SYNJ1
TANGO2
TARS2
TIMM22
TIMM50
TK2
TMEM70
TRMT5
TRMU
TSFM
TTC26
TUFM
TWNK
UQCC3
UQCRB
UQCRC2
UQCRQ
WARS2
YARS1
YARS2

LMRGs, lactate metabolism related genes.

Table S2. Description of key genes.

ID	description	logFC	AveExpr	t	P.Value	B
FLI1	Fli-1 Proto-Oncogene, ETS Transcription Factor	0.967357665	8.62109208	4.548372862	3.11E-05	2.167779339
GATA2	GATA Binding Protein 2	-0.564169117	6.438372026	-3.106210991	0.003022462	-2.110275166
INPP5K	Inositol Polyphosphate-5-Phosphatase K	-0.285190787	9.932591634	-2.106309376	0.039856937	-4.403424567
KCNN4	Potassium Calcium-Activated Channel Subfamily N Member 4	1.133693153	9.255508158	5.155295071	3.72E-06	4.190581766
NDUFB3	NADH:Ubiquinone Oxidoreductase Subunit B3	0.321635908	10.75693215	2.37519718	0.021132742	-3.855093711
NDUFS3	NADH:Ubiquinone Oxidoreductase Core Subunit S3	0.437804676	11.17087945	3.948070612	0.000230463	0.27827737
PC	Pyruvate Carboxylase	-0.763079185	8.60136667	-3.19676283	0.002327676	-1.87096884
PIGA	Phosphatidylinositol Glycan Anchor Biosynthesis Class A	-1.135036805	7.766421476	-5.070992389	5.02E-06	3.904204148

SCO2	Synthesis Of Cytochrome C Oxidase 2	0.493001901	9.800014662	2.713872942	0.008915482	-3.090264897
SLC16A7	Solute Carrier Family 16 Member 7	-1.322559794	7.335392848	-4.585245082	2.74E-05	2.287821031
SLC25A4	Solute Carrier Family 25 Member 4	-0.600749717	8.694689156	-3.872185907	0.000294226	0.049220594
TCIRG1	T Cell Immune Regulator 1, ATPase H+ Transporting V0 Subunit A3	0.509815144	10.47379002	2.795209164	0.007174171	-2.895035711
TSFM	Ts Translation Elongation Factor, Mitochondrial	0.272946246	9.522463494	2.11406087	0.03916024	-4.388385734
UQCRQ	Ubiquinol-Cytochrome C Reductase Complex III Subunit VII	0.340375954	12.58818901	2.572341385	0.012894521	-3.419522182

Table S3. GO and KEGG enrichment analysis results.

ONTOL<br>OGY	ID	Description	Gene<br>Ratio	BgRat<br>io	pvalue	qvalue
BP	GO:000<br>6091	generation of precursor<br>metabolites and energy	6/14	494/1<br>8800	8.01678<br>E-07	0.00022<br>466
BP	GO:001<br>5980	energy derivation by oxidation of<br>organic compounds	5/14	321/1<br>8800	2.48181<br>E-06	0.00031<br>4362
BP	GO:002<br>2904	respiratory electron transport<br>chain	4/14	113/1<br>8800	1.18242<br>E-06	0.00022<br>466
CC	GO:000<br>5743	mitochondrial inner membrane	5/14	491/1<br>9594	1.60744<br>E-05	0.00028<br>348
CC	GO:009<br>8798	mitochondrial protein-containing<br>complex	4/14	281/1<br>9594	3.70066<br>E-05	0.00028<br>348
CC	GO:190<br>2495	transmembrane transporter<br>complex	4/14	377/1<br>9594	0.00011<br>5885	0.00050<br>5365
MF	GO:002<br>2804	active transmembrane transporter<br>activity	4/13	404/1<br>8410	0.00013<br>9615	0.00348<br>9348
MF	GO:000<br>8137	NADH dehydrogenase<br>(ubiquinone) activity	2/13	42/18<br>410	0.00039<br>0053	0.00348<br>9348
MF	GO:005<br>0136	NADH dehydrogenase (quinone)<br>activity	2/13	43/18<br>410	0.00040<br>8917	0.00348<br>9348
KEGG	hsa001<br>90	Oxidative phosphorylation	4/11	134/8<br>164	2.0945<br>E-05	0.00059<br>5278

GO, gene ontology. BP, biological process. CC, cellular component. MF, molecular function. KEGG, Kyoto encyclopedia of genes and genomes.

Table S4. GSEA results.

ID	setSize	enrichmentScore	NES	pvalue	qvalue
REACTOME_PI3K_EVENTS_IN_ERBB2_SIGNALING	14	-0.807348026	-2.099666578	2.07E-05	0.000823619
REACTOME_PI3K_EVENTS_IN_ERBB4_SIGNALING	8	-0.89937732	-1.997282166	4.07E-05	0.001236899
REACTOME_TNFR2_NON_CANONICAL_NF_KB_PATHWAY	83	0.490487666	1.841227968	0.000143833	0.003385929
PID_PI3KCI_PATHWAY	39	0.60815759	1.980975042	0.000186168	0.004193597
REACTOME_FCERI_MEDIATED_NF_KB_ACTIVATION	69	0.482902183	1.7559589	0.001052995	0.015643259
REACTOME_CONSTITUTIVE_SIGNALING_BY_AKT1_E17K_IN_CANCER	19	-0.662385617	-1.866015969	0.001639206	0.021415112
PID_IL12_STAT4_PATHWAY	31	0.593999191	1.841809742	0.001745397	0.022107033

REACTOME_FCERI_MEDIATED_MAPK_ACTIVATION	32	0.58497305	1.825944825	0.00180045	0.02290908
REACTOME_TNF_RECEPTOR_SUPERFAMILY_TNFSF_MEMBERS_MEDIATING_NON_CANONICAL_NF_KB_PATHWAY	13	0.732576088	1.832978016	0.002437609	0.028025978
REACTOME_AKT_PHOSPHORYLATES_TARGETS_IN_THE_NUCLEUS	9	-0.778881559	-1.788216732	0.003329248	0.035920838
REACTOME_PI3K_AKT_SIGNALING_IN_CANCER	85	-0.406547819	-1.584953856	0.004520693	0.043856425
WP_REGULATORY_CIRCUITS_OF_THE_STAT3_SIGNALING_PATHWAY	65	0.443962684	1.598475607	0.006990214	0.05881183
KEGG_TGF_BETA_SIGNALING_PATHWAY	74	-0.402307943	-1.531083089	0.009951918	0.070496067
WP_BMP2WNT4FOXO1_PATHWAY_IN_PRIMARY_ENDOMETRIAL_STROMAL_CELL_DIFFERENTIATION	11	-0.693473541	-1.685178651	0.012075363	0.079273105
WP_TGFBETA_SIGNALING_PATHWAY	116	-0.349938037	-1.435266793	0.013058403	0.082383856

PID_PI3KCI_AKT_PATHWAY	30	-0.518110998	-1.632876266	0.013273581	0.083069429
KEGG_MAPK_SIGNALING_PATHWAY	209	-0.303494851	-1.350417852	0.013468097	0.083485289
WP_HIPPO_SIGNALING_REGULATION_PATHWAYS	83	-0.378595135	-1.470446812	0.015749628	0.092223425
REACTOME_SIGNALING_BY_TGFB_FAMILY_MEMBERS	102	-0.35202522	-1.414862165	0.020075632	0.109689682
WP_MAPK_SIGNALING_PATHWAY	197	-0.298049515	-1.316445955	0.023711542	0.119095632
REACTOME_NF_KB_ACTIVATION_THROUGH_FADD_RIP_1_PATHWAY_MEDIATED_BY_CASPASE_8_AND_10	9	0.706919808	1.599811697	0.026624611	0.129309954
BIOCARTA_AKT_PATHWAY	21	-0.541991093	-1.565217141	0.027917447	0.131972301

WP_FACTORS_AND_PATHWAYS_AFFECTING_INSULINLIKE_GROWTH_FACTOR_IGF1AKT_SIGNALING	26	-0.505126435	-1.537050396	0.030050403	0.137500369
REACTOME_GENE_AND_PROTEIN_EXPRESSION_BY_JAK_STAT_SIGNALING_AFTER_INTERLEUKIN_12_STIMULATION	27	0.508604079	1.527310522	0.031947376	0.14235985
REACTOME_PI3K_AKT_ACTIVATION	9	-0.682641298	-1.567260871	0.034313725	0.149474842
REACTOME_ACTIVATED_NTRK3_SIGNALS_THROUGH_PI3K	6	-0.754698783	-1.54155991	0.035883329	0.153444113
REACTOME_WNT5A_DEPENDENT_INTERNALIZATION_OF_FZD4	12	0.638266592	1.562241326	0.036503586	0.155401787
REACTOME_TGF_BETA_RECEPTOR_SIGNALING_ACTIVATES_SMADS	38	-0.440013694	-1.46456348	0.038936106	0.161175168

GSEA, gene set enrichment analysis.

Table S5. GSVA results.

ONTOLOGY	logFC	AveExpr	t	P.Value	B
HALLMARK_ALLOGRAFT_REJECTION	0.45545616	-0.011525194	7.108047647	1.30E-10	13.76249507
HALLMARK_CHOLESTEROL_HOMEOSTASIS	-0.352819438	0.000137768	-5.515851772	2.37E-07	6.451036982
HALLMARK_INTERFERON_ALPHA_RESPONSE	0.397283402	-0.005514261	5.186667378	9.99E-07	5.060033144
HALLMARK_INTERFERON_GAMMA_RESPONSE	0.347563425	-0.006794682	4.947328009	2.76E-06	4.081006347
HALLMARK_COMPLEMENT	0.302433081	0.000124513	4.941530264	2.83E-06	4.05764672
HALLMARK_MYC_TARGETS_V2	-0.348253356	-0.013581995	-4.63892379	9.82E-06	2.862993862
HALLMARK_UV_RESPONSE_DN	-0.259778684	0.012602726	-4.254254309	4.46E-05	1.418259199
HALLMARK_INFLAMMATORY_RESPONSE	0.245311954	0.002894006	3.936345957	0.000146217	0.291957192
HALLMARK_IL6_JAK_STAT3_SIGNALING	0.236870655	-0.025771634	3.314970141	0.001245835	-1.714557928

HALLMARK_TNFA_SIGNALING_VIA_NFKB	-0.239258098	-0.021385709	-3.267192762	0.001453703	-1.857435367
HALLMARK_P53_PATHWAY	-0.186861859	0.019155147	-3.21288996	0.001729129	-2.017759557
HALLMARK_PROTEIN_SECRETION	0.198474581	0.018323632	2.978199708	0.003575463	-2.684865058
HALLMARK_ESTROGEN_RESPONSE_EARLY	-0.18651964	-0.018050312	-2.902052322	0.00448844	-2.892124113
HALLMARK_ADIPOGENESIS	-0.168162982	0.017337667	-2.724597443	0.00750331	-3.357186711
HALLMARK_MYOGENESIS	-0.172954165	-0.002719435	-2.607264008	0.010407449	-3.650636955
HALLMARK_UV_RESPONSE_UP	-0.145495162	-0.000565189	-2.393217492	0.018414171	-4.156433692
HALLMARK_UNFOLDED_PROTEIN_RESPONSE	-0.142284042	0.025029971	-2.160713833	0.032911349	-4.661499963
HALLMARK_PI3K_AKT_MTOR_SIGNALING	0.138051673	-0.008075765	2.140843144	0.034519835	-4.702480141

HALLMARK_HYPOXIA	-0.13096364	0.007227524	-2.094327843	0.038553557	-4.797049055
HALLMARK_REACTIVE_OXYGEN_SPECIES_PATHWAY	-0.128713204	0.008655306	-1.998021833	0.048209789	-4.986743032

GSVA, gene set variation analysis.

Table S6. mRNA-miRNA interactions.

mRNA	miRNA
FLI1	hsa-miR-429
FLI1	hsa-miR-493-5p
FLI1	hsa-miR-627-5p
FLI1	hsa-miR-653-5p
FLI1	hsa-miR-421
FLI1	hsa-miR-770-5p
FLI1	hsa-miR-216b-5p
GATA2	hsa-miR-25-3p
GATA2	hsa-miR-27a-3p
GATA2	hsa-miR-214-3p
GATA2	hsa-miR-128-3p
GATA2	hsa-miR-423-3p
GATA2	hsa-miR-520a-5p
GATA2	hsa-miR-769-5p
GATA2	hsa-miR-1286
GATA2	hsa-miR-3163
GATA2	hsa-miR-3612
GATA2	hsa-miR-378d
GATA2	hsa-miR-378f
GATA2	hsa-miR-378g
GATA2	hsa-miR-4731-5p
GATA2	hsa-miR-766-5p
KCNN4	hsa-miR-103a-3p
KCNN4	hsa-miR-195-5p
KCNN4	hsa-miR-1307-3p
NDUFS3	hsa-miR-125a-5p

PC	hsa-miR-30a-5p
PC	hsa-miR-96-5p
PC	hsa-miR-30b-5p
PC	hsa-miR-302a-3p
PIGA	hsa-miR-19a-3p
PIGA	hsa-miR-19b-3p
PIGA	hsa-miR-32-5p
PIGA	hsa-miR-30d-5p
PIGA	hsa-miR-448
PIGA	hsa-miR-520d-3p
PIGA	hsa-miR-3942-5p
SLC16A7	hsa-miR-29a-3p
SLC16A7	hsa-miR-153-3p
SLC16A7	hsa-miR-125a-5p
SLC16A7	hsa-miR-629-5p

Table S7. mRNA-TF interactions.

mRNA	miRNA
GATA2	GATA1
GATA2	RAD21
GATA2	USF1
GATA2	USF2
GATA2	CTCF
KCNN4	CTCF
KCNN4	FOS
KCNN4	FOSL2
KCNN4	JUN
KCNN4	JUND
KCNN4	RAD21
KCNN4	REST
KCNN4	SPI1
KCNN4	STAG1
NDUFB3	ERG
PC	CEBPB
PC	CTCF
PC	ELF1
PC	EP300
PC	ERG
PC	FOS
PC	FOSL2
PC	JUN
PC	MAX
PC	MYC
PC	NR3C1

PC	RAD21
PIGA	CEBPB
PIGA	CTCF
PIGA	FOXA1
PIGA	NR3C1
PIGA	RAD21
PIGA	STAG1
SCO2	CEBPA
SCO2	CEBPB
SCO2	CTCF
SCO2	MAX
SCO2	MYC
SCO2	NRF1
SCO2	SPI1
SCO2	USF2
TCIRG1	ESR1
TSFM	CTCF
UQCRQ	CTCF
UQCRQ	GABPA
UQCRQ	RAD21

TF, transcription factor.

Table S8. mRNA-drug interactions.

mRNA	drug
SLC25A4	pirinixic acid
SLC25A4	Ethinyl Estradiol
GATA2	Tetrachlorodibenzodioxin
PC	Benzo(a)pyrene
SCO2	pirinixic acid
SLC16A7	pirinixic acid
FLI1	bisphenol A
FLI1	Carbon Tetrachloride
FLI1	Tetrachlorodibenzodioxin
FLI1	titanium dioxide
GATA2	Benzo(a)pyrene
GATA2	Carbon Tetrachloride
GATA2	epoxiconazole
GATA2	Estradiol
GATA2	Nanotubes, Carbon
GATA2	pirinixic acid
GATA2	Valproic Acid
NDUFB3	bisphenol A
NDUFB3	pirinixic acid
NDUFB3	Tetrachlorodibenzodioxin
NDUFB3	titanium dioxide
PC	bisphenol A
PC	Dietary Fats
PC	epoxiconazole
PC	Ethinyl Estradiol
PC	Nanotubes, Carbon

PC	propiconazole
PC	Tetrachlorodibenzodioxin
PIGA	Benzo(a)pyrene
PIGA	Carbon Tetrachloride
PIGA	Ethylnitrosourea
SCO2	Tetrachlorodibenzodioxin
SCO2	titanium dioxide
SLC16A7	Phenobarbital
SLC16A7	Tetrachlorodibenzodioxin
SLC25A4	bisphenol A
SLC25A4	Carbon Tetrachloride
SLC25A4	Dibutyl Phthalate
SLC25A4	Tetrachlorodibenzodioxin

Table S9. mRNA-RBP interactions.

mRNA	RBP
FLI1	U2AF2
GATA2	ALYREF
GATA2	FUS
GATA2	YTHDC1
KCNN4	ALYREF
KCNN4	RNPS1
NDUFB3	ELAVL1
NDUFS3	ALYREF
NDUFS3	IGF2BP1
NDUFS3	IGF2BP3
NDUFS3	RNPS1
NDUFS3	SCAF4
NDUFS3	SCAF8
NDUFS3	YBX1
NDUFS3	YTHDF1
PC	HNRNPK
PC	PCBP2
PC	PTBP1
PC	SRSF1
PC	UPF1
PIGA	ALYREF
SLC16A7	HNRNPL
SLC16A7	TARDBP
TCIRG1	HNRNPC
TCIRG1	HNRNPK
TCIRG1	U2AF1

TSFM	ALYREF
TSFM	TARDBP
TSFM	YTHDF1

RBP, RNA binding protein.

Data_Figure S3

gene	sensitivity	specificity
FLI1	1	0
FLI1	0.916667	0
FLI1	0.916667	0.083333
FLI1	0.833333	0.083333
FLI1	0.75	0.083333
FLI1	0.75	0.166667
FLI1	0.75	0.25
FLI1	0.75	0.333333
FLI1	0.75	0.416667
FLI1	0.666667	0.416667
FLI1	0.666667	0.5
FLI1	0.666667	0.583333
FLI1	0.583333	0.583333
FLI1	0.5	0.583333
FLI1	0.416667	0.583333
FLI1	0.333333	0.583333
FLI1	0.333333	0.666667
FLI1	0.25	0.666667
FLI1	0.25	0.75
FLI1	0.166667	0.75
FLI1	0.166667	0.833333
FLI1	0.083333	0.833333
FLI1	0.083333	0.916667
FLI1	0.083333	1
FLI1	0	1
GATA2	1	0
GATA2	1	0.083333
GATA2	0.916667	0.083333
GATA2	0.916667	0.166667
GATA2	0.916667	0.25
GATA2	0.833333	0.25
GATA2	0.833333	0.333333
GATA2	0.75	0.333333
GATA2	0.75	0.416667
GATA2	0.666667	0.416667
GATA2	0.583333	0.416667
GATA2	0.5	0.416667
GATA2	0.5	0.5
GATA2	0.5	0.583333
GATA2	0.5	0.666667
GATA2	0.416667	0.666667
GATA2	0.333333	0.666667

GATA2	0.333333	0.75
GATA2	0.25	0.75
GATA2	0.166667	0.75
GATA2	0.083333	0.75
GATA2	0	0.75
GATA2	0	0.833333
GATA2	0	0.916667
GATA2	0	1
INPP5K	1	0
INPP5K	0.916667	0
INPP5K	0.916667	0.083333
INPP5K	0.833333	0.083333
INPP5K	0.833333	0.166667
INPP5K	0.833333	0.25
INPP5K	0.75	0.25
INPP5K	0.75	0.333333
INPP5K	0.75	0.416667
INPP5K	0.666667	0.416667
INPP5K	0.666667	0.5
INPP5K	0.666667	0.583333
INPP5K	0.666667	0.666667
INPP5K	0.583333	0.666667
INPP5K	0.5	0.666667
INPP5K	0.416667	0.666667
INPP5K	0.416667	0.75
INPP5K	0.416667	0.833333
INPP5K	0.333333	0.833333
INPP5K	0.333333	0.916667
INPP5K	0.333333	1
INPP5K	0.25	1
INPP5K	0.166667	1
INPP5K	0.083333	1
INPP5K	0	1
KCNN4	1	0
KCNN4	1	0.083333
KCNN4	1	0.166667
KCNN4	1	0.25
KCNN4	0.916667	0.25
KCNN4	0.916667	0.333333
KCNN4	0.916667	0.416667
KCNN4	0.916667	0.5
KCNN4	0.833333	0.5
KCNN4	0.833333	0.583333
KCNN4	0.75	0.583333

KCNN4	0.75	0.666667
KCNN4	0.75	0.75
KCNN4	0.75	0.833333
KCNN4	0.75	0.916667
KCNN4	0.666667	0.916667
KCNN4	0.583333	0.916667
KCNN4	0.5	0.916667
KCNN4	0.416667	0.916667
KCNN4	0.333333	0.916667
KCNN4	0.25	0.916667
KCNN4	0.25	1
KCNN4	0.166667	1
KCNN4	0.083333	1
KCNN4	0	1
NDUFB3	1	0
NDUFB3	0.916667	0
NDUFB3	0.833333	0
NDUFB3	0.75	0
NDUFB3	0.75	0.083333
NDUFB3	0.75	0.166667
NDUFB3	0.666667	0.166667
NDUFB3	0.583333	0.166667
NDUFB3	0.583333	0.25
NDUFB3	0.583333	0.333333
NDUFB3	0.583333	0.416667
NDUFB3	0.583333	0.5
NDUFB3	0.583333	0.583333
NDUFB3	0.5	0.583333
NDUFB3	0.416667	0.583333
NDUFB3	0.333333	0.583333
NDUFB3	0.333333	0.666667
NDUFB3	0.25	0.666667
NDUFB3	0.25	0.75
NDUFB3	0.166667	0.75
NDUFB3	0.166667	0.833333
NDUFB3	0.083333	0.833333
NDUFB3	0.083333	0.916667
NDUFB3	0.083333	1
NDUFB3	0	1
NDUFS3	1	0
NDUFS3	1	0.083333
NDUFS3	1	0.166667
NDUFS3	0.916667	0.166667
NDUFS3	0.916667	0.25

NDUFS3	0.916667	0.416667
NDUFS3	0.916667	0.5
NDUFS3	0.833333	0.5
NDUFS3	0.75	0.5
NDUFS3	0.666667	0.5
NDUFS3	0.583333	0.5
NDUFS3	0.5	0.5
NDUFS3	0.5	0.583333
NDUFS3	0.5	0.666667
NDUFS3	0.416667	0.666667
NDUFS3	0.416667	0.75
NDUFS3	0.333333	0.75
NDUFS3	0.25	0.75
NDUFS3	0.166667	0.75
NDUFS3	0.166667	0.833333
NDUFS3	0.166667	0.916667
NDUFS3	0.083333	0.916667
NDUFS3	0	0.916667
NDUFS3	0	1
PC	1	0
PC	1	0.083333
PC	1	0.166667
PC	1	0.25
PC	1	0.333333
PC	1	0.416667
PC	0.916667	0.416667
PC	0.833333	0.416667
PC	0.833333	0.5
PC	0.75	0.5
PC	0.666667	0.5
PC	0.583333	0.5
PC	0.5	0.5
PC	0.416667	0.5
PC	0.416667	0.583333
PC	0.416667	0.666667
PC	0.333333	0.666667
PC	0.25	0.666667
PC	0.166667	0.666667
PC	0.083333	0.666667
PC	0	0.666667
PC	0	0.75
PC	0	0.833333
PC	0	0.916667
PC	0	1

PIGA	1	0
PIGA	1	0.083333
PIGA	1	0.166667
PIGA	0.916667	0.166667
PIGA	0.833333	0.166667
PIGA	0.75	0.166667
PIGA	0.75	0.25
PIGA	0.75	0.333333
PIGA	0.75	0.416667
PIGA	0.666667	0.416667
PIGA	0.666667	0.5
PIGA	0.583333	0.5
PIGA	0.583333	0.583333
PIGA	0.583333	0.666667
PIGA	0.5	0.666667
PIGA	0.416667	0.666667
PIGA	0.416667	0.75
PIGA	0.333333	0.75
PIGA	0.333333	0.833333
PIGA	0.25	0.833333
PIGA	0.166667	0.833333
PIGA	0.083333	0.833333
PIGA	0.083333	0.916667
PIGA	0.083333	1
PIGA	0	1
SCO2	1	0
SCO2	1	0.083333
SCO2	0.916667	0.083333
SCO2	0.916667	0.166667
SCO2	0.916667	0.25
SCO2	0.916667	0.333333
SCO2	0.833333	0.333333
SCO2	0.833333	0.416667
SCO2	0.75	0.416667
SCO2	0.75	0.5
SCO2	0.666667	0.5
SCO2	0.666667	0.583333
SCO2	0.583333	0.583333
SCO2	0.583333	0.666667
SCO2	0.583333	0.75
SCO2	0.583333	0.833333
SCO2	0.583333	0.916667
SCO2	0.583333	1
SCO2	0.5	1

SCO2	0.416667	1
SCO2	0.333333	1
SCO2	0.25	1
SCO2	0.166667	1
SCO2	0.083333	1
SCO2	0	1
SLC16A7	1	0
SLC16A7	1	0.083333
SLC16A7	1	0.166667
SLC16A7	0.916667	0.166667
SLC16A7	0.833333	0.166667
SLC16A7	0.75	0.166667
SLC16A7	0.75	0.25
SLC16A7	0.666667	0.25
SLC16A7	0.583333	0.25
SLC16A7	0.583333	0.333333
SLC16A7	0.583333	0.416667
SLC16A7	0.583333	0.5
SLC16A7	0.5	0.5
SLC16A7	0.5	0.583333
SLC16A7	0.5	0.666667
SLC16A7	0.5	0.75
SLC16A7	0.5	0.833333
SLC16A7	0.5	0.916667
SLC16A7	0.416667	0.916667
SLC16A7	0.333333	0.916667
SLC16A7	0.25	0.916667
SLC16A7	0.166667	0.916667
SLC16A7	0.083333	1
SLC16A7	0	1
SLC25A4	1	0
SLC25A4	1	0.083333
SLC25A4	0.916667	0.083333
SLC25A4	0.916667	0.166667
SLC25A4	0.916667	0.25
SLC25A4	0.833333	0.25
SLC25A4	0.75	0.25
SLC25A4	0.666667	0.25
SLC25A4	0.666667	0.333333
SLC25A4	0.666667	0.416667
SLC25A4	0.583333	0.416667
SLC25A4	0.5	0.416667
SLC25A4	0.5	0.5
SLC25A4	0.5	0.583333

SLC25A4	0.5	0.666667
SLC25A4	0.416667	0.666667
SLC25A4	0.333333	0.666667
SLC25A4	0.25	0.666667
SLC25A4	0.166667	0.666667
SLC25A4	0.083333	0.666667
SLC25A4	0.083333	0.75
SLC25A4	0.083333	0.833333
SLC25A4	0.083333	0.916667
SLC25A4	0	0.916667
SLC25A4	0	1
TCIRG1	1	0
TCIRG1	1	0.083333
TCIRG1	1	0.25
TCIRG1	1	0.333333
TCIRG1	1	0.416667
TCIRG1	1	0.5
TCIRG1	1	0.583333
TCIRG1	0.916667	0.583333
TCIRG1	0.833333	0.583333
TCIRG1	0.833333	0.666667
TCIRG1	0.75	0.666667
TCIRG1	0.666667	0.666667
TCIRG1	0.666667	0.75
TCIRG1	0.583333	0.75
TCIRG1	0.5	0.75
TCIRG1	0.416667	0.75
TCIRG1	0.416667	0.833333
TCIRG1	0.333333	0.833333
TCIRG1	0.25	0.833333
TCIRG1	0.166667	0.833333
TCIRG1	0.166667	0.916667
TCIRG1	0.083333	0.916667
TCIRG1	0	0.916667
TCIRG1	0	1
TSFM	1	0
TSFM	1	0.083333
TSFM	0.916667	0.083333
TSFM	0.833333	0.083333
TSFM	0.833333	0.166667
TSFM	0.75	0.166667
TSFM	0.75	0.25
TSFM	0.666667	0.25
TSFM	0.666667	0.333333

TSFM	0.666667	0.416667
TSFM	0.666667	0.5
TSFM	0.583333	0.5
TSFM	0.583333	0.583333
TSFM	0.5	0.583333
TSFM	0.416667	0.583333
TSFM	0.333333	0.583333
TSFM	0.333333	0.666667
TSFM	0.333333	0.75
TSFM	0.25	0.75
TSFM	0.25	0.833333
TSFM	0.25	0.916667
TSFM	0.166667	0.916667
TSFM	0.083333	0.916667
TSFM	0	0.916667
TSFM	0	1
UQCRQ	1	0
UQCRQ	1	0.083333
UQCRQ	1	0.166667
UQCRQ	1	0.25
UQCRQ	0.916667	0.25
UQCRQ	0.833333	0.25
UQCRQ	0.833333	0.333333
UQCRQ	0.833333	0.416667
UQCRQ	0.75	0.416667
UQCRQ	0.75	0.5
UQCRQ	0.75	0.583333
UQCRQ	0.666667	0.583333
UQCRQ	0.666667	0.666667
UQCRQ	0.666667	0.75
UQCRQ	0.583333	0.833333
UQCRQ	0.583333	0.916667
UQCRQ	0.5	0.916667
UQCRQ	0.416667	0.916667
UQCRQ	0.333333	0.916667
UQCRQ	0.25	0.916667
UQCRQ	0.166667	1
UQCRQ	0.083333	1
UQCRQ	0	1

Data_Figure S7

gene	sensitivity	specificity
FLI1	1	0
FLI1	1	0.058824
FLI1	1	0.117647
FLI1	1	0.176471
FLI1	1	0.235294
FLI1	1	0.294118
FLI1	1	0.352941
FLI1	1	0.411765
FLI1	1	0.470588
FLI1	0.857143	0.470588
FLI1	0.857143	0.529412
FLI1	0.857143	0.588235
FLI1	0.714286	0.588235
FLI1	0.571429	0.588235
FLI1	0.428571	0.588235
FLI1	0.428571	0.647059
FLI1	0.285714	0.647059
FLI1	0.285714	0.705882
FLI1	0.142857	0.705882
FLI1	0.142857	0.764706
FLI1	0.142857	0.823529
FLI1	0.142857	0.882353
FLI1	0.142857	0.941176
FLI1	0	0.941176
FLI1	0	1
GATA2	1	0
GATA2	1	0.058824
GATA2	1	0.117647
GATA2	1	0.176471
GATA2	1	0.235294
GATA2	1	0.294118
GATA2	1	0.352941
GATA2	1	0.411765
GATA2	1	0.470588
GATA2	0.857143	0.470588
GATA2	0.857143	0.529412
GATA2	0.857143	0.588235
GATA2	0.857143	0.647059
GATA2	0.857143	0.705882
GATA2	0.857143	0.764706
GATA2	0.857143	0.823529
GATA2	0.857143	0.882353

GATA2	0.714286	0.882353
GATA2	0.571429	0.882353
GATA2	0.571429	0.941176
GATA2	0.428571	0.941176
GATA2	0.285714	0.941176
GATA2	0.285714	1
GATA2	0.142857	1
GATA2	0	1
INPP5K	1	0
INPP5K	1	0.058824
INPP5K	1	0.117647
INPP5K	1	0.176471
INPP5K	1	0.235294
INPP5K	0.857143	0.235294
INPP5K	0.714286	0.235294
INPP5K	0.714286	0.294118
INPP5K	0.714286	0.352941
INPP5K	0.714286	0.411765
INPP5K	0.714286	0.470588
INPP5K	0.571429	0.470588
INPP5K	0.571429	0.529412
INPP5K	0.571429	0.588235
INPP5K	0.571429	0.647059
INPP5K	0.571429	0.705882
INPP5K	0.428571	0.705882
INPP5K	0.428571	0.764706
INPP5K	0.428571	0.823529
INPP5K	0.285714	0.823529
INPP5K	0.285714	0.882353
INPP5K	0.285714	0.941176
INPP5K	0.142857	0.941176
INPP5K	0	0.941176
INPP5K	0	1
KCNN4	1	0
KCNN4	1	0.058824
KCNN4	1	0.117647
KCNN4	1	0.176471
KCNN4	1	0.235294
KCNN4	1	0.294118
KCNN4	1	0.352941
KCNN4	1	0.411765
KCNN4	1	0.470588
KCNN4	1	0.529412
KCNN4	1	0.588235

KCNN4	1	0.647059
KCNN4	1	0.705882
KCNN4	0.857143	0.705882
KCNN4	0.714286	0.705882
KCNN4	0.714286	0.764706
KCNN4	0.571429	0.764706
KCNN4	0.571429	0.823529
KCNN4	0.428571	0.823529
KCNN4	0.428571	0.882353
KCNN4	0.285714	0.882353
KCNN4	0.142857	0.882353
KCNN4	0.142857	0.941176
KCNN4	0	0.941176
KCNN4	0	1
NDUFB3	1	0
NDUFB3	1	0.058824
NDUFB3	1	0.117647
NDUFB3	1	0.176471
NDUFB3	1	0.235294
NDUFB3	1	0.294118
NDUFB3	1	0.352941
NDUFB3	0.857143	0.352941
NDUFB3	0.857143	0.411765
NDUFB3	0.857143	0.470588
NDUFB3	0.714286	0.470588
NDUFB3	0.714286	0.529412
NDUFB3	0.714286	0.588235
NDUFB3	0.714286	0.647059
NDUFB3	0.571429	0.647059
NDUFB3	0.428571	0.647059
NDUFB3	0.428571	0.705882
NDUFB3	0.285714	0.705882
NDUFB3	0.142857	0.705882
NDUFB3	0.142857	0.764706
NDUFB3	0.142857	0.823529
NDUFB3	0.142857	0.882353
NDUFB3	0.142857	0.941176
NDUFB3	0	0.941176
NDUFB3	0	1
NDUFS3	1	0
NDUFS3	1	0.058824
NDUFS3	1	0.117647
NDUFS3	1	0.176471
NDUFS3	1	0.235294

NDUFS3	1	0.294118
NDUFS3	1	0.352941
NDUFS3	1	0.411765
NDUFS3	0.857143	0.411765
NDUFS3	0.857143	0.470588
NDUFS3	0.857143	0.529412
NDUFS3	0.714286	0.529412
NDUFS3	0.714286	0.588235
NDUFS3	0.571429	0.588235
NDUFS3	0.428571	0.588235
NDUFS3	0.428571	0.647059
NDUFS3	0.428571	0.705882
NDUFS3	0.428571	0.764706
NDUFS3	0.428571	0.823529
NDUFS3	0.428571	0.941176
NDUFS3	0.428571	1
NDUFS3	0.285714	1
NDUFS3	0.142857	1
NDUFS3	0	1
PC	1	0
PC	1	0.058824
PC	1	0.117647
PC	1	0.176471
PC	1	0.235294
PC	1	0.294118
PC	0.857143	0.294118
PC	0.857143	0.352941
PC	0.714286	0.352941
PC	0.714286	0.411765
PC	0.571429	0.411765
PC	0.571429	0.470588
PC	0.571429	0.529412
PC	0.571429	0.588235
PC	0.571429	0.647059
PC	0.428571	0.647059
PC	0.285714	0.647059
PC	0.142857	0.647059
PC	0.142857	0.705882
PC	0.142857	0.764706
PC	0.142857	0.823529
PC	0.142857	0.882353
PC	0.142857	0.941176
PC	0.142857	1
PC	0	1

PIGA	1	0
PIGA	1	0.058824
PIGA	1	0.117647
PIGA	1	0.176471
PIGA	1	0.235294
PIGA	1	0.294118
PIGA	1	0.352941
PIGA	1	0.411765
PIGA	1	0.470588
PIGA	1	0.529412
PIGA	1	0.588235
PIGA	1	0.647059
PIGA	1	0.705882
PIGA	1	0.764706
PIGA	1	0.823529
PIGA	1	0.882353
PIGA	0.857143	0.882353
PIGA	0.714286	0.882353
PIGA	0.714286	0.941176
PIGA	0.714286	1
PIGA	0.571429	1
PIGA	0.428571	1
PIGA	0.285714	1
PIGA	0.142857	1
PIGA	0	1
SCO2	1	0
SCO2	1	0.058824
SCO2	1	0.117647
SCO2	0.857143	0.117647
SCO2	0.857143	0.176471
SCO2	0.857143	0.235294
SCO2	0.714286	0.235294
SCO2	0.714286	0.294118
SCO2	0.714286	0.352941
SCO2	0.571429	0.352941
SCO2	0.571429	0.411765
SCO2	0.571429	0.470588
SCO2	0.571429	0.529412
SCO2	0.428571	0.529412
SCO2	0.285714	0.529412
SCO2	0.142857	0.529412
SCO2	0	0.529412
SCO2	0	0.588235
SCO2	0	0.647059

SCO2	0	0.705882
SCO2	0	0.764706
SCO2	0	0.823529
SCO2	0	0.882353
SCO2	0	0.941176
SCO2	0	1
SLC16A7	1	0
SLC16A7	1	0.058824
SLC16A7	1	0.176471
SLC16A7	1	0.235294
SLC16A7	1	0.294118
SLC16A7	1	0.352941
SLC16A7	1	0.411765
SLC16A7	1	0.470588
SLC16A7	1	0.529412
SLC16A7	1	0.588235
SLC16A7	1	0.647059
SLC16A7	1	0.705882
SLC16A7	1	0.764706
SLC16A7	1	0.823529
SLC16A7	1	0.882353
SLC16A7	1	0.941176
SLC16A7	1	1
SLC16A7	0.857143	1
SLC16A7	0.714286	1
SLC16A7	0.571429	1
SLC16A7	0.428571	1
SLC16A7	0.285714	1
SLC16A7	0.142857	1
SLC16A7	0	1
SLC25A4	1	0
SLC25A4	1	0.058824
SLC25A4	1	0.117647
SLC25A4	1	0.176471
SLC25A4	1	0.235294
SLC25A4	1	0.294118
SLC25A4	1	0.352941
SLC25A4	1	0.411765
SLC25A4	0.857143	0.411765
SLC25A4	0.857143	0.470588
SLC25A4	0.857143	0.529412
SLC25A4	0.714286	0.529412
SLC25A4	0.714286	0.588235
SLC25A4	0.714286	0.647059

SLC25A4	0.571429	0.647059
SLC25A4	0.571429	0.705882
SLC25A4	0.428571	0.705882
SLC25A4	0.428571	0.764706
SLC25A4	0.428571	0.823529
SLC25A4	0.285714	0.823529
SLC25A4	0.142857	0.823529
SLC25A4	0	0.823529
SLC25A4	0	0.882353
SLC25A4	0	0.941176
SLC25A4	0	1
TCIRG1	1	0
TCIRG1	1	0.058824
TCIRG1	1	0.176471
TCIRG1	1	0.235294
TCIRG1	1	0.294118
TCIRG1	1	0.352941
TCIRG1	0.857143	0.352941
TCIRG1	0.857143	0.411765
TCIRG1	0.857143	0.470588
TCIRG1	0.857143	0.529412
TCIRG1	0.857143	0.588235
TCIRG1	0.857143	0.647059
TCIRG1	0.857143	0.705882
TCIRG1	0.714286	0.705882
TCIRG1	0.714286	0.764706
TCIRG1	0.714286	0.823529
TCIRG1	0.714286	0.882353
TCIRG1	0.714286	0.941176
TCIRG1	0.714286	1
TCIRG1	0.571429	1
TCIRG1	0.428571	1
TCIRG1	0.285714	1
TCIRG1	0.142857	1
TCIRG1	0	1
TSFM	1	0
TSFM	1	0.058824
TSFM	1	0.117647
TSFM	1	0.176471
TSFM	1	0.235294
TSFM	1	0.294118
TSFM	1	0.352941
TSFM	0.857143	0.352941
TSFM	0.857143	0.411765

TSFM	0.714286	0.411765
TSFM	0.571429	0.411765
TSFM	0.571429	0.470588
TSFM	0.571429	0.529412
TSFM	0.571429	0.588235
TSFM	0.428571	0.588235
TSFM	0.428571	0.647059
TSFM	0.428571	0.705882
TSFM	0.428571	0.764706
TSFM	0.428571	0.823529
TSFM	0.428571	0.882353
TSFM	0.428571	0.941176
TSFM	0.428571	1
TSFM	0.285714	1
TSFM	0.142857	1
TSFM	0	1
UQCRQ	1	0
UQCRQ	1	0.058824
UQCRQ	1	0.117647
UQCRQ	1	0.176471
UQCRQ	1	0.235294
UQCRQ	1	0.294118
UQCRQ	1	0.352941
UQCRQ	1	0.411765
UQCRQ	1	0.470588
UQCRQ	1	0.529412
UQCRQ	1	0.588235
UQCRQ	1	0.647059
UQCRQ	1	0.705882
UQCRQ	1	0.764706
UQCRQ	0.857143	0.823529
UQCRQ	0.714286	0.823529
UQCRQ	0.714286	0.882353
UQCRQ	0.714286	0.941176
UQCRQ	0.714286	1
UQCRQ	0.571429	1
UQCRQ	0.285714	1
UQCRQ	0.142857	1
UQCRQ	0	1
